# Supplementary material for: Empirical methods for controlling false positives and estimating confidence in ChIP-Seq peaks
Source: BMC Bioinformatics. 2008 Dec 5;9:523. doi: 10.1186/1471-2105-9-523 (PMC2628906; doi:10.1186/1471-2105-9-523)
Supplement: Additional File 1 — A variety of html documents from the USeq web site detailing the available applications, their best usage, output file type descriptions, command line menus, etc. [file 1471-2105-9-523-S1.zip › USeqUserGuides/JavaDoc.zip!/JavaDoc/index-files/index-18.html]

 
 
 
 
 
 
S-Index
 


 

 
function windowTitle()
{
    parent.document.title="S-Index";
}
 
 
 

 

 


 
   
  
 
 
 
   
 
   
          Overview     
        Package   
        Class   
        Use   
          Tree     
          Deprecated     
       Index    
          Help     
   
 
 
  
 
 
 

 
  
   PREV LETTER   
   NEXT LETTER    
  
    FRAMES     
   NO FRAMES     
  
    All Classes  ');
  }
  //-->
 
 
    All Classes  
 


  
 
 
  
 

 A   B   C   D   E   F   G   H   I   J   L   M   N   O   P   Q   R   S   T   U   V   W   X   Z   
    
 S  
 
   S3CodeSamples   - Class in  util.amazon      S3CodeSamples()   - 
Constructor for class util.amazon. S3CodeSamples 
  
   S3Manager   - Class in  util.amazon  Class to handle Amazon S3 file upload and download using the JetS3t API.   S3Manager(String, String, File)   - 
Constructor for class util.amazon. S3Manager 
 Constructor, after instantiating check that boolean.connected is true;
   s3Service   - 
Variable in class util.amazon. S3Manager 
  
   sam(float[], float[], double)   - 
Static method in class util.gen. Num 
 Calculates the SAM d statistic, similar to a t-statistic with a bit of fudge added into the denominator to
 control for zero variance conditions.
   SamChromosomeData   - Class in  trans.main      SamChromosomeData(int, int)   - 
Constructor for class trans.main. SamChromosomeData 
  
   Sample   - Class in  edu.utah.seq.methylation454      Sample(String)   - 
Constructor for class edu.utah.seq.methylation454. Sample 
  
   SAMPLES_PROPERTIES_NAME   - 
Static variable in class util.amazon. S3CodeSamples 
  
   save1lq()   - 
Method in class trans.misc. ConvertAgilentData 
  
   saveAlignerPreferences()   - 
Method in class gata.aligner. AlignerPreferences 
  
   saveAndNullQuantiles()   - 
Method in class trans.cel. QuantileNormalization 
 Takes the Quantile[][], fetches the float[] values for each, nulls the Quantile[], saves the float[] to disk.
   saveArrayList(File, ArrayList)   - 
Static method in class gata.main. GATAUtil 
  
   saveBinaryBooleanArray(boolean[], File)   - 
Method in class util.bio.seq. BinaryBooleanReaderWriter 
 Saves a boolean[] in a compressed binary format.
   saveBufferedImage(double, File, boolean)   - 
Method in class trans.graphics. IntervalDrawPanel 
  
   saveBufferedImage(double, File, boolean)   - 
Method in class trans.graphics. RankedSetDrawPanel 
  
   saveBufferedImage(double, File)   - 
Method in class util.bio.cluster. ClusterDrawPanel 
 Saves a png for this panel, don't paint(g2), this requires a head and will throw a headless error.
   saveGFF3TextFile(Gff3Feature[], File)   - 
Static method in class util.bio.parsers.gff. Gff3Feature 
 Prints an array of GFF3Features as a plain text file
   SaveImageForm   - Class in  gata.menu      SaveImageForm(GATAParams)   - 
Constructor for class gata.menu. SaveImageForm 
  
   SaveImagePanel   - Class in  gata.menu      SaveImagePanel(GATAParams, JFrame)   - 
Constructor for class gata.menu. SaveImagePanel 
  
   saveIntensities()   - 
Method in class trans.cel. QuantileNormalization 
 Saves intensities, one float[] for each replica.
   saveMarkers(DBUtil, boolean)   - 
Method in class bioroot.plasmid. PlasmidBean 
  
   saveMarkers(DBUtil, boolean)   - 
Method in class bioroot.strain. StrainBean 
  
   saveMatrixValues(float[][], int[][], float, File)   - 
Static method in class util.gen. Num 
 Given a square table and a list of coordinates, save the associated values.
   saveNewAntibodyPreps(DBUtil)   - 
Method in class bioroot.antibody. AntibodyBean 
  
   saveNewUseDilutions(DBUtil)   - 
Method in class bioroot.antibody. AntibodyPrep 
  
   saveObject(File, Object)   - 
Static method in class gata.main. GATAUtil 
  
   saveObject(File, Object)   - 
Static method in class util.gen. IO 
  
   saveParentIds(DBUtil, boolean)   - 
Method in class bioroot.strain. StrainBean 
 Can delete old strainIds from StrainParent table, and add new entries if any are
 present in the parentConcats.
   savePlasmidIds(DBUtil, boolean)   - 
Method in class bioroot.antibody. AntibodyBean 
 Can delete old plasmidIds from AntibodyPlasmid table, and add new entries if any are
 present in the plasmidConcats.
   savePlasmidIds(DBUtil, boolean)   - 
Method in class bioroot.strain. StrainBean 
 Can delete old plasmidIds from StrainPlasmid table, and add new entries if any are
 present in the plasmidConcats.
   savePNG()   - 
Method in class util.bio.cluster. HierarchicalClustering 
  
   savePointAndHeatMapData(File, int, int, SmoothingWindow[], Info, boolean)   - 
Method in class edu.utah.seq.analysis. ScanSeqs 
 Saves point and heat map bar files of a given score.
   saveRegionsAsCoordinatesArray(File, Region[])   - 
Static method in class trans.anno. RandomRegions 
 Writes the chrom start stop of a Region[] as a binary file.
   saveScores(File)   - 
Static method in class trans.roc. ExtractSaveSgrScores 
  
   saveSmoothedHeatMapData(int, SmoothingWindow[], Info, File, String, String, boolean)   - 
Method in class edu.utah.seq.analysis. ScanSeqs 
  
   saveSmoothedPointData(int, SmoothingWindow[], Info, File, String, String)   - 
Method in class edu.utah.seq.analysis. ScanSeqs 
  
   saveStrainIds(DBUtil, boolean)   - 
Method in class bioroot.antibody. AntibodyBean 
 Can delete old strainIds from AntibodyStrain table, and add new entries if any are
 present in the strainConcats.
   saveStrainIds(DBUtil, boolean)   - 
Method in class bioroot.plasmid. PlasmidBean 
 Can delete old strainIds from PlasmidStrain table, and add new entries if any are
 present in the strainConcats.
   saveTPMap()   - 
Method in class trans.misc. ConvertAgilentData 
  
   saveTPMap()   - 
Method in class trans.misc. ConvertNimblegenNDF2TPMap 
  
   saveTPMap(TPMapLine[], File)   - 
Static method in class trans.tpmap. TPMapLine 
  
   saveXMLDocument(String, Document)   - 
Method in class util.xml. ParseXMLFile 
 Saves XML Document into XML file.
   scalar(double, SplineScalar)   - 
Method in class trans.cel. SplineScalar 
 Use to calculate a scaler that when multiplied by the testValue brings the testValue up to the model.
   scale1To1000(float[])   - 
Static method in class util.gen. Num 
 Scales the array to 1:1000
   ScaleBar   - Class in  gata.geneGlyphs      ScaleBar(AnnoSpecParams, GATAParams)   - 
Constructor for class gata.geneGlyphs. ScaleBar 
  
   scaleQuantileArraysByControlIntensities()   - 
Method in class trans.cel. QuantileNormalization 
 Scales a quantile array that is sorted by original position in the MapFeature[] 
 using the median value of the control intensities.
   scaleQuantileArraysByMedian(Quantile[][], float)   - 
Static method in class trans.cel. QuantileNormalization 
 Takes arrays of Quantile that have been sorted by value, and scales their values to the target median.
   scaleValuesPositions()   - 
Method in class trans.graphics. Graph 
  
   scanBarDirectories()   - 
Method in class trans.misc. ConvertBars2TPMapCel 
  
   ScanChip   - Class in  trans.main  Scores windows of intensity values using several tests.   ScanChip(String[])   - 
Constructor for class trans.main. ScanChip 
  
   ScanChromosomes   - Class in  trans.main  Scores windows of intensity values using several tests.   ScanChromosomes(String[])   - 
Constructor for class trans.main. ScanChromosomes 
  
   scanChromosomes()   - 
Method in class trans.main. T2 
  
   scanChromosomes()   - 
Method in class util.apps. ScoreChromosomes 
  
   ScanChromosomesCNV   - Class in  trans.main  Scores windows of intensity values using several tests.   ScanChromosomesCNV(String[])   - 
Constructor for class trans.main. ScanChromosomesCNV 
  
   scanEm(String[], String[], double)   - 
Method in class meme. MotifScanner 
 Scans a set of sequences for the motif, saves results in a MemeResults object.
   ScanGenes   - Class in  trans.main  Given a list of genes and their exons extracts the associated intensities.   ScanGenes(String[])   - 
Constructor for class trans.main. ScanGenes 
  
   scanIntervals()   - 
Method in class trans.main. ScoreIntervals 
  
   scanPrintAllSequences(String[], String[])   - 
Method in class meme. MotifScanner 
 Scans a set of sequences for the motif, prints as it goes.
   scanPrintNumberHits(double, String[], String[])   - 
Method in class meme. MotifScanner 
 Scans a set of sequences for the motif, prints as it goes.
   scanPrintSequences(String[], String[], double)   - 
Method in class meme. MotifScanner 
 Scans a set of sequences for the motif, prints as it goes.
   ScanSeqs   - Class in  edu.utah.seq.analysis  Takes PointData and generates sliding window scan statistics.   ScanSeqs(String[])   - 
Constructor for class edu.utah.seq.analysis. ScanSeqs 
  
   scanSequence(String)   - 
Method in class meme. MotifScanner 
 Scans a sequence for the motif, scores every window, forward and reverse complement, assigning the 
 higher score to the center base.
   scanSgrArray()   - 
Method in class trans.roc. SgrBacCounter 
 Scans an sgr array, sorting into bins.
   ScatterDrawFrame   - Class in  util.apps  Helper class for   ScatterPlot  , the frame.   ScatterDrawFrame(String[])   - 
Constructor for class util.apps. ScatterDrawFrame 
  
   ScatterDrawPanel   - Class in  util.apps  Helper class for   ScatterPlot  , the guts of drawing the scatter plot.   ScatterDrawPanel(String[])   - 
Constructor for class util.apps. ScatterDrawPanel 
  
   ScatterPlot   - Class in  util.apps  Draws a simple scatter plot and calculates a Pearson correlation coefficient 
 for two serialized int[] or float[] arrays.   ScatterPlot()   - 
Constructor for class util.apps. ScatterPlot 
  
   SCORE_DESCRIPTIONS   - 
Static variable in class edu.utah.seq.analysis. ScanSeqs 
  
   SCORE_ID   - 
Static variable in class edu.utah.seq.analysis. ScanSeqs 
  
   SCORE_NAMES   - 
Static variable in class edu.utah.seq.analysis. ScanSeqs 
  
   SCORE_UNITS   - 
Static variable in class edu.utah.seq.analysis. ScanSeqs 
  
   scoreBlastResults()   - 
Method in class util.bio.parsers. BlastCompompositeGenerator 
  
   ScoreChromosomes   - Class in  util.apps  Scores chromosomes for the presence of transcription factor binding sites.   ScoreChromosomes(String[])   - 
Constructor for class util.apps. ScoreChromosomes 
  
   scoreConsensus(int[][], String, float)   - 
Method in class edu.utah.seq.parsers. ElandSequenceParser 
  
   ScoredCoordinate   - Class in  util.bio.annotation      ScoredCoordinate(String, int, int, double)   - 
Constructor for class util.bio.annotation. ScoredCoordinate 
  
   ScoredGene   - Class in  trans.main      ScoredGene(UCSCGeneLine, float[][], float[][])   - 
Constructor for class trans.main. ScoredGene 
  
   ScoreDNAAlignment(String, String, int, int, int, int)   - 
Static method in class util.bio.calc. Alignment 
 Will score a DNA alignment given the two sequences, and scoring parameters.
   ScoredRegionsFormatFixer   - Class in  trans.misc  Converts a file format to something usable by Excel from the joinRegionsWithScores.sh script.   ScoredRegionsFormatFixer()   - 
Constructor for class trans.misc. ScoredRegionsFormatFixer 
  
   scoreGroup(int, char[], char[], char[])   - 
Method in class util.bio.parsers. BlastCompompositeGenerator 
  
   ScoreIntervals   - Class in  trans.main  Scores Intervals for the presence of transcription factor binding sites.   ScoreIntervals(String[])   - 
Constructor for class trans.main. ScoreIntervals 
  
   scoreIt(boolean)   - 
Method in class bioroot. SearchHit 
 Weighting boundary matches as a score of 5, loose matches as 2.
   scoreLines(String[])   - 
Method in class util.bio.parsers. BlastCompompositeGenerator 
  
   scorePairedControls()   - 
Method in class edu.utah.seq.analysis. ScanSeqs 
 Scanner for generating the null control vs control data.
   ScoreParsedBars   - Class in  trans.roc  Returns values that overlap particular regions, can generate p-values associated with random back ground model   ScoreParsedBars(File, int, int)   - 
Constructor for class trans.roc. ScoreParsedBars 
  
   ScoreParsedBars(String[])   - 
Constructor for class trans.roc. ScoreParsedBars 
  
   scoreSequence(double, String)   - 
Method in class meme. MotifScanner 
 Returns an array of MotifHits that are > or = to the loglikelihood cutOff.
   ScoreSequences   - Class in  util.apps  Scores sequences for the presence of transcription factor binding sites.   ScoreSequences(String[])   - 
Constructor for class util.apps. ScoreSequences 
  
   scoresToString()   - 
Method in class util.bio.parsers. UCSCGeneLine 
  
   scoreSubSeq(String)   - 
Method in class meme. MotifScanner 
 ignores non GATC chars
   scoreTreatments()   - 
Method in class edu.utah.seq.analysis. PeakPicker 
 Used for window scanning treatment only datasets
   scoreTreatmentsControls()   - 
Method in class edu.utah.seq.analysis. PeakPicker 
 Used for window scanning with both treatment and control data.
   scoreWindow(int)   - 
Method in class trans.main. ScanChip 
 Scores a window using a pseudo median or trimmed mean on oligo relative differences.
   scoreWindow(int)   - 
Method in class trans.main. ScanChromosomes 
 Scores a window using a pseudo median or trimmed mean on oligo relative differences.
   scoreWindow(int)   - 
Method in class trans.main. ScanChromosomesCNV 
 Scores a window using a pseudo median or trimmed mean on oligo relative differences.
   Search   - Class in  bioroot      Search()   - 
Constructor for class bioroot. Search 
  
   SearchBean   - Class in  bioroot  Bean to hold info about a search request   SearchBean()   - 
Constructor for class bioroot. SearchBean 
  
   searchDataFiles()   - 
Method in class meme. MemeR 
  
   searchForSimilarMatches(String[], String[], DBUtil, boolean, UserBean)   - 
Method in class bioroot. SearchBean 
 Searches a list of columns for a set of words, case insensitive, %word% matches.
   searchForXamers(String[], String[][])   - 
Static method in class util.bio.xamer. XamerCounter 
 Creates an ArrayList of Xamer objects, where each Xamer contains all the seqs, by round
 that were found to match a particular xamers[i]
   searchHeadings   - 
Static variable in class bioroot.antibody. AntibodyBase 
 Use to make a hash of column heading in which to search, name then the actual search column name.
   searchHeadings   - 
Static variable in class bioroot.oligo. OligoBase 
 Use to make a hash of column heading in which to search, name then the actual search column name.
   searchHeadings   - 
Static variable in class bioroot.plasmid. PlasmidBase 
 Use to make a hash of column heading in which to search, name then the actual search column name.
   searchHeadings   - 
Static variable in class bioroot.strain. StrainBase 
 Use to make a hash of column heading in which to search, name then the actual search column name.
   SearchHit   - Class in  bioroot  Holds info relating to a search hit from a database LIKE query.   SearchHit(String, String, int, String, boolean)   - 
Constructor for class bioroot. SearchHit 
  
   segment()   - 
Method in class trans.misc. CalculateGeneExtensions 
  
   Segment   - Class in  util.bio.wrappers  This wraps Huber's segment() function in his 'tilingArray' R package.   Segment(File, File, File)   - 
Constructor for class util.bio.wrappers. Segment 
  
   segment()   - 
Method in class util.bio.wrappers. Segment 
  
   segmentGenes()   - 
Method in class trans.misc. CalculateGeneExtensions 
  
   segmentGrArray(Gr[])   - 
Method in class trans.misc. CalculateGeneExtensions 
  
   selectHitsToChromosomes()   - 
Method in class edu.utah.seq.data. Simulator 
 Randomly picks chromosomes for generating points weighted by file size.
   selex   - package selex For processing of raw SELEX sequence data.   SelexParams   - Class in  selex  Hold parameters and results generated by the   SelexSeqParser  ; also generates reports.   SelexParams()   - 
Constructor for class selex. SelexParams 
  
   SelexSeqParser   - Class in  selex  Parses out oligos from multiple rounds of SELEX.   SelexSeqParser()   - 
Constructor for class selex. SelexSeqParser 
  
   Seq   - Class in  util.bio.seq  For manipulating nucleic acid sequences.   Seq()   - 
Constructor for class util.bio.seq. Seq 
  
   SeqFiles   - Class in  selex  Continer for raw sequence information, seqFile, qualFile, and the actual reads from each.   SeqFiles(String, String, SelexParams)   - 
Constructor for class selex. SeqFiles 
  
   SeqRead   - Class in  selex  Container for information related to a particular sequencing read.   SeqRead(String, String, String, String, SelexParams, SeqFiles)   - 
Constructor for class selex. SeqRead 
  
   Seqs   - Class in  gata.aligner      Seqs(AlignParams, MultiFastaParser, MultiFastaParser, int)   - 
Constructor for class gata.aligner. Seqs 
  
   serialVersionUID   - 
Static variable in class edu.utah.seq.data. EnrichedRegion 
  
   serialVersionUID   - 
Static variable in class edu.utah.seq.data. Info 
  
   serialVersionUID   - 
Static variable in class edu.utah.seq.data. SmoothingWindow 
  
   serialVersionUID   - 
Static variable in class edu.utah.seq.data. SmoothingWindowInfo 
  
   serialVersionUID   - 
Static variable in class trans.tpmap. IntensityFeature 
  
   serialVersionUID   - 
Static variable in class trans.tpmap. MapFeature 
  
   setA(double)   - 
Method in class gata.main. GATAParams 
  
   setA(int)   - 
Method in class util.bio.parsers. BaseCount 
  
   setAAlignPanel(AlignPanel)   - 
Method in class gata.main. GATAParams 
  
   setAbsoluteDifference(int)   - 
Method in class trans.main. WilcoxonSamplePair 
  
   setAddOneLLPSPMCutOffScore(double)   - 
Method in class meme. MotifSearchResult 
  
   setAffiliation(String)   - 
Method in class cgar.beans. LabGroupBean 
  
   setAlias(String)   - 
Method in class bioroot. ReagentBean 
  
   setAlias(String)   - 
Method in class util.bio.parsers.gff. Gff3Feature 
  
   setAlignFrameWidth(int)   - 
Method in class gata.plotter. AlignPanel 
  
   setAlignment(String[])   - 
Method in class util.bio.wrappers. BlastResult 
  
   setAlignmentEnd(int)   - 
Method in class edu.utah.seq.methylation454. Read 
  
   setAlignmentStart(int)   - 
Method in class edu.utah.seq.methylation454. Read 
  
   setAlignPanel(AlignPanel)   - 
Method in class gata.main. GATAParams 
  
   setAlignPanel(AlignPanel)   - 
Method in class gata.menu. GATAFrame 
  
   setAlignParams(AlignParams)   - 
Method in class gata.main. GATAParams 
  
   setAmplicons(Amplicon[])   - 
Method in class edu.utah.seq.methylation454. Sample 
  
   setAnnoPanelHeight(int)   - 
Method in class gata.main. AnnoSpecParams 
  
   setAnnoScaleDefaults(AnnoSpecParams)   - 
Method in class gata.main. PlotterPreferences 
  
   setAnnotation(Annotation)   - 
Method in class gata.main. AnnoSpecParams 
  
   setAnnotationBackground()   - 
Method in class gata.geneGlyphs. Annotation 
  
   setAntibodyId(int)   - 
Method in class bioroot.antibody. AntibodyPrep 
  
   setAntibodyPreference(Preference)   - 
Method in class bioroot. UserBean 
  
   setAntibodyPreps(AntibodyPrep[])   - 
Method in class bioroot.antibody. AntibodyBean 
  
   setAntibodyUse(String)   - 
Method in class bioroot.antibody. UseDilution 
  
   setAntigen(String)   - 
Method in class bioroot.antibody. AntibodyBean 
  
   setAnyGenericGlyphs(boolean)   - 
Method in class gata.main. AnnoSpecParams 
  
   setAppDescription(String)   - 
Method in class util.apps.gump. Gump 
  
   setAppDescriptionS(JTextArea)   - 
Method in class util.apps.gump. Gump 
  
   setApplicationPanel(GumpApplicationPanel)   - 
Method in class util.apps.gump. GumpDrawFrame 
  
   setAppName(JTextArea)   - 
Method in class util.apps.gump. Gump 
  
   setArchived(boolean)   - 
Method in class bioroot. LabGroup 
  
   setARFA(RankedFloatArray)   - 
Method in class util.gen. SpearmanCorrelation 
  
   setArrayType(String)   - 
Method in class bioroot.strain. StrainBean 
  
   setAvailability(String)   - 
Method in class bioroot. ReagentBean 
  
   setAwsCredentials(AWSCredentials)   - 
Method in class util.amazon. S3Manager 
  
   setB(double)   - 
Method in class gata.main. GATAParams 
  
   setBackgroundColor(Color)   - 
Method in class gata.main. GATAParams 
  
   setBarDirectory(File)   - 
Method in class trans.main. WindowBlockMakerTwoColor 
  
   setBarFile(File)   - 
Method in class edu.utah.seq.parsers. BarParser 
  
   setBase(char)   - 
Method in class util.bio.parsers. BaseCount 
  
   setBasePositions(int[])   - 
Method in class edu.utah.seq.parsers. BarParser 
  
   setBasePositions(int[])   - 
Method in class trans.misc. GrGraph 
  
   setBaseScore(float)   - 
Method in class edu.utah.seq.data. HeatMapMaker 
  
   setBaseScore(float)   - 
Method in class trans.main. WindowBlockMaker 
  
   setBaseScores(double[])   - 
Method in class trans.main. Interval 
  
   setBathrooms(double)   - 
Method in class util.apps. HouseBean 
  
   setBedrooms(int)   - 
Method in class util.apps. HouseBean 
  
   setBestSubWindow(SmoothingWindow)   - 
Method in class edu.utah.seq.data. EnrichedRegion 
  
   setBestSubWindow(SubWindow)   - 
Method in class trans.main. Interval 
  
   setBestWindow(SmoothingWindow)   - 
Method in class edu.utah.seq.data. EnrichedRegion 
  
   setBestWindow(Window)   - 
Method in class trans.main. Interval 
  
   setBestWindowScored(boolean)   - 
Method in class trans.main. Interval 
  
   setBiggestAlignPanelY(int)   - 
Method in class gata.main. GATAParams 
  
   setBiggestGlyphPanelY(int)   - 
Method in class gata.main. AnnoSpecParams 
  
   setBindingPeaks(BindingPeak[])   - 
Method in class trans.main. Interval 
  
   setBioroot(DBUtil)   - 
Method in class bioroot. UserRegistrationBean 
  
   setBl2SeqHits(ArrayList)   - 
Method in class bioroot. BlastMatcher 
  
   setBl2SeqProg(String)   - 
Method in class gata.aligner. AlignerPreferences 
  
   setBlasted(boolean)   - 
Method in class bioroot.plasmid. PlasmidBean 
  
   setBlastResults(String)   - 
Method in class bioroot. SearchBean 
  
   setBooleanArray(boolean[], boolean)   - 
Static method in class gata.main. GATAUtil 
 Takes an array of boolean[] and sets all to the boolean value
   setBottomCluster(Cluster)   - 
Method in class util.bio.cluster. Cluster 
  
   setBpMatches(String)   - 
Method in class bioroot.plasmid. OligoMatch 
  
   setBRFA(RankedFloatArray)   - 
Method in class util.gen. SpearmanCorrelation 
  
   setBucket(S3Bucket)   - 
Method in class util.amazon. S3Manager 
  
   setBucketName(String)   - 
Method in class util.amazon. S3Manager 
  
   setButton(JButton, int, int)   - 
Static method in class gata.main. GATAUtil 
  
   setButton(JButton, int, int)   - 
Static method in class util.gen. Swing 
  
   setC(double)   - 
Method in class gata.main. GATAParams 
  
   setC(int)   - 
Method in class util.bio.parsers. BaseCount 
  
   setCdsEnd(int)   - 
Method in class util.bio.parsers. UCSCGeneLine 
  
   setCdsStart(int)   - 
Method in class util.bio.parsers. UCSCGeneLine 
  
   setCelFiles(File[])   - 
Method in class trans.main. Interval 
  
   setCellTypeId(int)   - 
Method in class bioroot.strain. StrainBean 
  
   setCellTypeName(String)   - 
Method in class bioroot.strain. StrainBean 
  
   setCelMasker(CelMasker)   - 
Method in class trans.graphics. VirtualCelFrame 
  
   setChallenge(String)   - 
Method in class bioroot. LoginBean 
  
   setChallengeDate(String)   - 
Method in class bioroot. LoginBean 
  
   setChartPanel(ChartPanel)   - 
Method in class trans.graphics. RankedSetAnalysis 
  
   setCheckMax(boolean)   - 
Method in class trans.qc. StatFlag 
  
   setCheckMin(boolean)   - 
Method in class trans.qc. StatFlag 
  
   setChipSetDirectories(File[])   - 
Method in class trans.main. ChromSet 
  
   setChooser(JFileChooser, int, int, int)   - 
Static method in class gata.main. GATAUtil 
  
   setChromosome(String)   - 
Method in class edu.utah.seq.data. EnrichedRegion 
  
   setChromosome(String)   - 
Method in class edu.utah.seq.data. Info 
  
   setChromosome(String)   - 
Method in class edu.utah.seq.methylation454. Reference 
  
   setChromosome(String)   - 
Method in class edu.utah.seq.parsers. BarParser 
  
   setChromosome(String)   - 
Method in class edu.utah.seq.parsers. ElandExportLine 
  
   setChromosome(String)   - 
Method in class trans.anno. BindingRegion 
  
   setChromosome(String)   - 
Method in class trans.anno. Region 
  
   setChromosome(String)   - 
Method in class trans.main. Interval 
  
   setChromosome(String)   - 
Method in class trans.main. PosRegion 
  
   setChromosome(String)   - 
Method in class trans.misc. GrGraph 
  
   setChromosome(String)   - 
Method in class trans.roc. RocWindow 
  
   setChromosome(String)   - 
Method in class util.bio.annotation. Coordinate 
  
   setChromosomeNameAppender(String)   - 
Method in class util.bio.parsers.gff. DmelRel4Extractor 
  
   setChromosomeNames(String[])   - 
Method in class cgar.beans. GenomeBean 
  
   setCity(String)   - 
Method in class util.apps. HouseBean 
  
   setClosestBindingRegion(BindingRegion)   - 
Method in class trans.anno. BindingRegion 
  
   setClosestInterval(Interval)   - 
Method in class trans.main. PosRegion 
  
   setClosestNonOverlappingInterval(int)   - 
Method in class trans.main. PosRegion 
  
   setColor(Color)   - 
Method in class gata.geneGlyphs. GenericGlyph 
  
   setColumnName(String)   - 
Method in class bioroot. Feature 
  
   setComments(String)   - 
Method in class bioroot. ReagentBean 
  
   setCompAnnoSpecParams(AnnoSpecParams)   - 
Method in class gata.main. GATAParams 
  
   setCompAnnotation(Annotation)   - 
Method in class gata.menu. GATAFrame 
 needed to add later since annotation doesn't exist when ToolFrame is created
   setCompGlyphPanel(GlyphPanel)   - 
Method in class gata.menu. GATAFrame 
  
   setComplete(boolean)   - 
Method in class bioroot. CellTypeBean 
  
   setComplete(boolean)   - 
Method in class bioroot. GeneBean 
  
   setComplete(boolean)   - 
Method in class bioroot. MarkerBean 
  
   setComplete(boolean)   - 
Method in class bioroot.oligo. OligoUploadBean 
  
   setComplete(boolean)   - 
Method in class bioroot. OrganismBean 
  
   setComplete(boolean)   - 
Method in class bioroot.plasmid. PlasmidUploadBean 
  
   setComplete(boolean)   - 
Method in class bioroot. ReagentBean 
  
   setComplete(boolean)   - 
Method in class bioroot.strain. StrainUploadBean 
  
   setComplete(boolean)   - 
Method in class bioroot. UserRegistrationBean 
  
   setCompVisible(boolean)   - 
Method in class gata.aligner. Alignment 
  
   setCompVisible(boolean)   - 
Method in class gata.aligner. AlignParams 
  
   setConcatStart(int)   - 
Method in class bioroot. BlastMatcher 
  
   setConcatStop(int)   - 
Method in class bioroot. BlastMatcher 
  
   setConcentration(String)   - 
Method in class bioroot.oligo. OligoBean 
  
   setConcentration(String)   - 
Method in class bioroot.plasmid. PlasmidBean 
  
   setConcentrationUnits(String)   - 
Method in class bioroot.oligo. OligoBean 
  
   setConnected(boolean)   - 
Method in class util.amazon. S3Manager 
  
   setConsole(Console)   - 
Method in class gata.geneGlyphs. GlyphPanel 
  
   setConsole(Console)   - 
Method in class gata.main. GATAParams 
  
   setConsole(Console)   - 
Method in class gata.plotter. AlignPanel 
  
   setContactEmail(String)   - 
Method in class bioroot. ContactBean 
  
   setControlChromosomeNames(String[])   - 
Method in class trans.cel. QuantileNormalization 
  
   setControlIndexes(int[][])   - 
Method in class trans.cel. QuantileNormalization 
  
   setControlPoints(Point[])   - 
Method in class edu.utah.seq.data. EnrichedRegion 
  
   setCorrelationCoefficient(double)   - 
Method in class util.bio.cluster. Cluster 
  
   setCpGs(CpG[])   - 
Method in class edu.utah.seq.methylation454. Amplicon 
  
   setCredentialsFile(File)   - 
Method in class util.amazon. S3Manager 
  
   setCurrentPlotterPreferences(GATAParams)   - 
Method in class gata.main. PlotterPreferences 
 Contains all the default preferences for GATAParams
   setCustomAttributes(String[])   - 
Method in class util.bio.parsers.gff. Gff3Feature 
  
   setD(double)   - 
Method in class gata.main. GATAParams 
  
   setDasCompatible(boolean)   - 
Method in class cgar.beans. ProcessedDataBean 
  
   setDate(String)   - 
Method in class bioroot.antibody. AntibodyPrep 
  
   setDate(String)   - 
Method in class cgar.beans. AnalysisBean 
  
   setDateLastLogin(String)   - 
Method in class cgar.beans. UserBean 
  
   setDbxref(String)   - 
Method in class util.bio.parsers.gff. Gff3Feature 
  
   setDefaultTrackThickness()   - 
Method in class gata.geneGlyphs. Annotation 
  
   setDefaultTrackThickness(float)   - 
Method in class gata.main. GATAParams 
  
   setDeletions(int)   - 
Method in class util.bio.parsers. BaseCount 
  
   setDemoResponse(String)   - 
Method in class bioroot. LoginBean 
  
   setDescription(String)   - 
Method in class cgar.beans. AnalysisBean 
  
   setDescription(String)   - 
Method in class cgar.beans. AnalysisGroupBean 
  
   setDescription(String)   - 
Method in class cgar.beans. AnalysisTypeBean 
  
   setDescription(String)   - 
Method in class cgar.beans. ProcessedDataTypeBean 
  
   setDescription(String)   - 
Method in class util.apps.gump. Gump 
  
   setDescription(String)   - 
Method in class util.apps.gump. Option 
  
   setDescriptionS(JTextArea)   - 
Method in class util.apps.gump. Option 
  
   setDifferentUserConcat(String)   - 
Method in class bioroot. UserRegistrationBean 
  
   setDilution(String)   - 
Method in class bioroot.antibody. UseDilution 
  
   setDisplayClusterPlot(boolean)   - 
Method in class util.bio.cluster. HierarchicalClustering 
  
   setDisplayName(String)   - 
Method in class util.bio.parsers. UCSCGeneLine 
  
   setDNAColor(Color)   - 
Method in class gata.main. GATAParams 
  
   setDNAVis(boolean)   - 
Method in class gata.main. GATAParams 
  
   setDrawCompBox(boolean)   - 
Method in class gata.plotter. AlignPanel 
  
   setDrawParams()   - 
Method in class gata.geneGlyphs. GlyphPanel 
 Fetches glyphParams to speed up drawing, call this to initialize variables forGlyphPanel
   setE(double)   - 
Method in class gata.main. GATAParams 
  
   setEditHistory(String)   - 
Method in class bioroot. CellTypeBean 
  
   setEditHistory(String)   - 
Method in class bioroot. GeneBean 
  
   setEditHistory(String)   - 
Method in class bioroot. MarkerBean 
  
   setEditHistory(String)   - 
Method in class bioroot. OrganismBean 
  
   setEditHistory(String)   - 
Method in class bioroot. ReagentBean 
  
   setEditHistory(String)   - 
Method in class bioroot. UserBean 
  
   setEFF_M(double)   - 
Method in class gata.aligner. AlignParams 
  
   setEFF_N(double)   - 
Method in class gata.aligner. AlignParams 
  
   setEmail(String)   - 
Method in class bioroot. UserBean 
  
   setEmail(String)   - 
Method in class bioroot. UserRegistrationBean 
  
   setEmail(String)   - 
Method in class cgar.beans. UserBean 
  
   setEmailAddresses(String[])   - 
Method in class bioroot. LabGroup 
  
   setEnd(int)   - 
Method in class trans.anno. Region 
  
   setEnd(int)   - 
Method in class trans.main. PosRegion 
  
   setEnd(int)   - 
Method in class trans.roc. RocWindow 
  
   setEnd(int)   - 
Method in class util.bio.annotation. ExonIntron 
  
   setEnd(int)   - 
Method in class util.bio.parsers.gff. Gff3Feature 
  
   setEndSet(boolean)   - 
Method in class util.bio.parsers.gff. Gff3Feature 
  
   setExistingMember(boolean)   - 
Method in class bioroot. UserRegistrationBean 
  
   setExons(ExonIntron[])   - 
Method in class util.bio.parsers. UCSCGeneLine 
  
   setExpect(String)   - 
Method in class util.bio.wrappers. BlastResult 
  
   setExpectation(double)   - 
Method in class util.bio.wrappers. BL2Seq 
  
   setEXTRACT(boolean)   - 
Method in class gata.aligner. AlignParams 
  
   setF(double)   - 
Method in class gata.main. GATAParams 
  
   setFailedLogins(int)   - 
Method in class bioroot. UserBean 
  
   setFamilyRooms(int)   - 
Method in class util.apps. HouseBean 
  
   setFastaFound(boolean)   - 
Method in class util.bio.parsers. MultiFastaParser 
  
   setFdr(double)   - 
Method in class trans.roc. Bin 
  
   setFeatures(Feature[])   - 
Method in class bioroot. Preference 
  
   setField(JTextField, int, int, int)   - 
Static method in class gata.main. GATAUtil 
  
   setField(JTextField, int, int, int)   - 
Static method in class util.gen. Swing 
  
   setFieldGroup(String)   - 
Method in class bioroot. SearchBean 
  
   setFields(String[])   - 
Method in class bioroot. SearchBean 
  
   setFileBaseName(String)   - 
Method in class meme. MemeResults 
  
   setFileName(String)   - 
Method in class bioroot.antibody. AntibodyBean 
  
   setFileName(String)   - 
Method in class bioroot.antibody. AntibodyPrep 
  
   setFileName(String)   - 
Method in class bioroot.oligo. OligoUploadBean 
  
   setFileName(String)   - 
Method in class bioroot.plasmid. PlasmidBean 
  
   setFileName(String)   - 
Method in class bioroot.plasmid. PlasmidUploadBean 
  
   setFileName(String)   - 
Method in class bioroot.strain. StrainBean 
  
   setFileName(String)   - 
Method in class bioroot.strain. StrainUploadBean 
  
   setFileNumber(int)   - 
Method in class meme. MotifSearchResult 
  
   setFirstName(String)   - 
Method in class bioroot. UserBean 
  
   setFirstName(String)   - 
Method in class bioroot. UserRegistrationBean 
  
   setFirstName(String)   - 
Method in class cgar.beans. UserBean 
  
   setFlag(String)   - 
Method in class util.apps.gump. Option 
  
   setFlagS(JTextArea)   - 
Method in class util.apps.gump. Option 
  
   setFloatArray(float[], float)   - 
Static method in class gata.main. GATAUtil 
 Takes an array of float[] and sets all to the float value
   setFloatArrayFile(File)   - 
Method in class util.bio.cluster. Cluster 
  
   setFractionIntersections(double[])   - 
Method in class trans.main. Interval 
  
   setFullName(String)   - 
Method in class bioroot. TableHeading 
  
   setFullPathToBL2seq(String)   - 
Method in class util.bio.wrappers. BL2Seq 
  
   setG(int)   - 
Method in class util.bio.parsers. BaseCount 
  
   setGap(String)   - 
Method in class util.bio.parsers.gff. Gff3Feature 
  
   setGapExtensionCost(int)   - 
Method in class util.bio.wrappers. BL2Seq 
  
   setGaps(String)   - 
Method in class util.bio.wrappers. BlastResult 
  
   setGarage(int)   - 
Method in class util.apps. HouseBean 
  
   setGataFrame(GATAFrame)   - 
Method in class gata.main. GATAParams 
  
   setGataPlotter(GATAPlotter)   - 
Method in class gata.main. GATAParams 
  
   setGcContent(double)   - 
Method in class trans.anno. BindingRegion 
  
   setGcContent(boolean[])   - 
Method in class trans.anno. Region 
  
   setGeneGlyphs(GeneRepGlyph[])   - 
Method in class gata.main. AnnoSpecParams 
  
   setGeneId(int)   - 
Method in class bioroot.antibody. AntibodyBean 
  
   setGeneId(int)   - 
Method in class bioroot.oligo. OligoBean 
  
   setGeneId(int)   - 
Method in class bioroot.plasmid. PlasmidBean 
  
   setGeneLines(UCSCGeneLine[])   - 
Method in class util.bio.parsers. UCSCGeneModelTableReader 
  
   setGeneName(String)   - 
Method in class bioroot.antibody. AntibodyBean 
  
   setGeneName(String)   - 
Method in class bioroot.oligo. OligoBean 
  
   setGeneName(String)   - 
Method in class bioroot.plasmid. PlasmidBean 
  
   setGenericLabelsVis(boolean)   - 
Method in class gata.main. GATAParams 
  
   setGeneSequence(String)   - 
Method in class bioroot.oligo. OligoBean 
  
   setGenomeId(int)   - 
Method in class cgar.beans. AnalysisBean 
  
   setGenomeVersion(String)   - 
Method in class trans.misc. GrGraph 
  
   setGenomicSequenceDirectory(File)   - 
Method in class trans.anno. RandomRegions 
  
   setGenomicSequenceParams(String)   - 
Method in class trans.main. LoadIntervalOligoInfo 
 Loads up the fasta file for a particular chromosome and sets some params.
   setGenomicSequenceParams(Interval)   - 
Method in class trans.main. ScoreIntervals 
 Loads up the fasta file for a particular chromosome and sets some params.
   setGenomicSequenceParams()   - 
Method in class trans.roc. PWMScoreWindows 
 Loads up the fasta file for a particular chromosome and sets some params.
   setGenotype(String)   - 
Method in class bioroot.strain. StrainBean 
  
   setGffCompPresent(boolean)   - 
Method in class gata.main. GATAParams 
  
   setGffFile(File)   - 
Method in class gata.main. AnnoSpecParams 
  
   setGFFFile(File)   - 
Method in class util.bio.parsers.gff. Gff3Parser 
  
   setGffRefPresent(boolean)   - 
Method in class gata.main. GATAParams 
  
   setGlyph(GenomicRegionGlyph)   - 
Method in class trans.graphics. GenomicRegion 
  
   setGlyphPanel(GlyphPanel)   - 
Method in class gata.main. AnnoSpecParams 
  
   setGraphics2DN(Graphics2D)   - 
Method in class gata.main. AnnoSpecParams 
  
   setGrowthConditions(String)   - 
Method in class bioroot.strain. StrainBean 
  
   setGuest(boolean)   - 
Method in class cgar.beans. UserBean 
  
   setH(double)   - 
Method in class gata.aligner. AlignParams 
  
   setHandlingInstructions(String)   - 
Method in class bioroot.oligo. OligoBean 
  
   setHeight(int)   - 
Method in class gata.main. GATAParams 
  
   setHeight(int)   - 
Method in class util.apps.gump. GumpDrawFrame 
  
   setHeightLabels(double)   - 
Method in class gata.main. GATAParams 
  
   setHits(String[])   - 
Method in class meme. MemeMotif 
  
   setHits(HashSet)   - 
Method in class trans.anno. Region 
  
   setHitsAddOneLLPSPM(ArrayList)   - 
Method in class meme. MotifSearchResult 
  
   setHitsMemeLLPSPM(ArrayList)   - 
Method in class meme. MotifSearchResult 
  
   setId(int)   - 
Method in class bioroot.antibody. AntibodyPrep 
  
   setId(int)   - 
Method in class bioroot.antibody. UseDilution 
  
   setId(int)   - 
Method in class bioroot. CellTypeBean 
  
   setId(int)   - 
Method in class bioroot. ContactBean 
  
   setId(int)   - 
Method in class bioroot. GeneBean 
  
   setId(int)   - 
Method in class bioroot. MarkerBean 
  
   setId(int)   - 
Method in class bioroot. OrganismBean 
  
   setId(int)   - 
Method in class bioroot. Organization 
  
   setId(int)   - 
Method in class bioroot.plasmid. OligoMatch 
  
   setId(int)   - 
Method in class bioroot. ReagentBean 
  
   setId(int)   - 
Method in class bioroot. UserBean 
  
   setId(int)   - 
Method in class cgar.beans. AnalysisBean 
  
   setId(int)   - 
Method in class cgar.beans. AnalysisGroupBean 
  
   setId(int)   - 
Method in class cgar.beans. AnalysisTypeBean 
  
   setId(int)   - 
Method in class cgar.beans. GenomeBean 
  
   setId(int)   - 
Method in class cgar.beans. LabGroupBean 
  
   setId(int)   - 
Method in class cgar.beans. ProcessedDataBean 
  
   setId(int)   - 
Method in class cgar.beans. ProcessedDataTypeBean 
  
   setId(int)   - 
Method in class cgar.beans. RawDataBean 
  
   setId(int)   - 
Method in class cgar.beans. SpeciesBean 
  
   setId(int)   - 
Method in class cgar.beans. UserBean 
  
   setId(int)   - 
Method in class cgar.beans. UserTypeBean 
  
   setId(int)   - 
Method in class cgar.beans. VisibilityBean 
  
   setId(String)   - 
Method in class util.bio.parsers.gff. Gff3Feature 
  
   setIdentities(String)   - 
Method in class util.bio.wrappers. BlastResult 
  
   setIncrement(double)   - 
Method in class util.gen. Histogram 
  
   setIndexedSequence(String)   - 
Method in class util.bio.seq. FetchIndexedSequence 
 Loads indexes for a particular chromosome.
   setInfo(Info)   - 
Method in class edu.utah.seq.data. InfoPoint 
  
   setInfo(Info)   - 
Method in class edu.utah.seq.data. PointData 
  
   setInfo(Info)   - 
Method in class edu.utah.seq.data. SmoothingWindowInfo 
  
   setInitialMessage()   - 
Method in class bioroot. ContactBean 
  
   setInitialTrackColors()   - 
Method in class gata.geneGlyphs. Annotation 
 Use to initialize all generic glyphs color field
   setInPeak(boolean)   - 
Method in class trans.main. Oligo 
  
   setInsertionLengths(ArrayList<Integer>)   - 
Method in class util.bio.parsers. BaseCount 
  
   setIntensities(float[][])   - 
Method in class trans.cel. QuantileNormalization 
  
   setIntensities(float[])   - 
Method in class trans.roc. ControlGene 
  
   setIntensityArrayLists(ArrayList)   - 
Method in class trans.bkgrnd. BackGroundOligo 
  
   setIntensityPM(double)   - 
Method in class trans.bkgrnd. LoadedOligo 
  
   setInterval(Interval)   - 
Method in class trans.main. PeakPicker 
  
   setJarFile(File)   - 
Method in class util.apps.gump. Gump 
  
   setJava(String)   - 
Method in class util.apps.gump. Gump 
  
   setJustPrint(boolean)   - 
Method in class util.bio.parsers. MultiFastaParser 
  
   setK(double)   - 
Method in class gata.aligner. AlignParams 
  
   setLabel(JLabel, int, int)   - 
Static method in class gata.main. GATAUtil 
  
   setLabel(JLabel, double, double)   - 
Static method in class util.bio.cluster. ClusterDrawPanel 
  
   setLabel(JLabel, int, int)   - 
Static method in class util.gen. Swing 
  
   setLabelColor(Color)   - 
Method in class gata.main. GATAParams 
  
   setLabelsVis(boolean)   - 
Method in class gata.main. GATAParams 
  
   setLabGroup(String)   - 
Method in class bioroot. LoginBean 
  
   setLabGroup(String)   - 
Method in class bioroot. UserBean 
  
   setLabGroup(String)   - 
Method in class bioroot. UserRegistrationBean 
  
   setLabGroupId(int)   - 
Method in class bioroot. ReagentBean 
  
   setLabGroupId(int)   - 
Method in class bioroot. UserBean 
  
   setLabGroupId(int)   - 
Method in class cgar.beans. AnalysisBean 
  
   setLabGroupName(String)   - 
Method in class bioroot. ContactBean 
  
   setLabGroupName(String)   - 
Method in class bioroot. ReagentBean 
  
   setLabGroups(String[])   - 
Method in class bioroot. Preference 
  
   setLabGroupUserTypes(LabGroupUserType[])   - 
Method in class cgar.beans. UserBean 
  
   setLAMBDA(double)   - 
Method in class gata.aligner. AlignParams 
  
   setLastArchiveDate(LabGroup[], DBUtil)   - 
Static method in class bioroot. CronArchive 
 If archived will set that labgroups lastArchive to today
   setLastLogin(String)   - 
Method in class bioroot. UserBean 
  
   setLastName(String)   - 
Method in class bioroot. UserBean 
  
   setLastName(String)   - 
Method in class bioroot. UserRegistrationBean 
  
   setLastName(String)   - 
Method in class cgar.beans. UserBean 
  
   setLastUser(String)   - 
Method in class bioroot.antibody. AntibodyPrep 
  
   setLastUser(String)   - 
Method in class bioroot. ReagentBean 
  
   setLatitude(double)   - 
Method in class util.apps. HouseBean 
  
   setLeftIndex(int)   - 
Method in class trans.main. PeakPicker 
  
   setLeftSlope(double)   - 
Method in class trans.main. Oligo 
  
   setLENGTH_COMPSEQ(int)   - 
Method in class gata.aligner. AlignParams 
  
   setLENGTH_REFSEQ(int)   - 
Method in class gata.aligner. AlignParams 
  
   setLine(Line2D)   - 
Method in class util.bio.cluster. Cluster 
  
   setLine(String)   - 
Method in class util.bio.wrappers. Primer3Pick 
  
   setLine2DArrays()   - 
Method in class gata.geneGlyphs. GlyphPanel 
 Call this to set or reset the array of Line2D[]'s representing the glyphs.
   setLocation(String)   - 
Method in class bioroot.antibody. AntibodyPrep 
  
   setLocation(String)   - 
Method in class bioroot. ReagentBean 
  
   setLoginName(String)   - 
Method in class bioroot. LoginBean 
  
   setLoginName(String)   - 
Method in class bioroot. UserBean 
  
   setLoginName(String)   - 
Method in class bioroot. UserRegistrationBean 
  
   setLoginName(String)   - 
Method in class cgar.beans. LoginBean 
  
   setLoginName(String)   - 
Method in class cgar.beans. UserBean 
  
   setLongitude(double)   - 
Method in class util.apps. HouseBean 
  
   setMakeChallenge(boolean)   - 
Method in class bioroot. LoginBean 
  
   setMakeFlattops(boolean)   - 
Method in class trans.main. PeakPicker 
  
   setMarkers(String[])   - 
Method in class bioroot.plasmid. PlasmidBean 
  
   setMarkers(String[])   - 
Method in class bioroot.strain. StrainBean 
  
   setMatchId(String)   - 
Method in class util.bio.wrappers. BlastResult 
  
   setMatching(String)   - 
Method in class bioroot. SearchBean 
  
   setMatchLength(String)   - 
Method in class util.bio.wrappers. BlastResult 
  
   setMatchName(String)   - 
Method in class util.bio.wrappers. BlastResult 
  
   setMatingType(String)   - 
Method in class bioroot.strain. StrainBean 
  
   setMaxBpGap(int)   - 
Method in class trans.main. PeakPicker 
  
   setMaxCluster(int)   - 
Method in class trans.main. Interval 
  
   setMaximum(double)   - 
Method in class trans.qc. StatFlag 
  
   setMaximum(double)   - 
Method in class util.gen. Histogram 
  
   setMaxNumFeatures(int)   - 
Method in class trans.tpmap. WindowMaker 
  
   setMaxNumPeaks(int)   - 
Method in class trans.main. PeakPicker 
  
   setMaxScore(double)   - 
Method in class gata.plotter. AlignPanel 
  
   setMaxScoreFractionDrop(double)   - 
Method in class trans.main. PeakPicker 
  
   setMaxValues()   - 
Method in class trans.misc. ConvertNimblegenPAIR2Cela 
  
   setMaxWindowFilter(float)   - 
Method in class edu.utah.seq.data. HeatMapMakerPosNeg 
  
   setMaxWindowFilter(double)   - 
Method in class trans.main. WindowBlockMakerTwoColor 
  
   setMedian(double)   - 
Method in class trans.main. ScoredGene 
  
   setMedian(double)   - 
Method in class trans.qc. StatFlag 
  
   setMedianScalar(double)   - 
Method in class trans.qc. CelFileStats 
  
   setMemeCmdLnArgs(String)   - 
Method in class meme. MemeResults 
  
   setMemeLLPSPMCutOffScore(double)   - 
Method in class meme. MotifSearchResult 
  
   setMemeParserArrayRef(MemeParser[])   - 
Method in class meme. MemeResults 
  
   setMemeParserRef(MemeParser)   - 
Method in class meme. MemeMotif 
  
   setMemePSPM(double[][])   - 
Method in class meme. MemeMotif 
  
   setMenu(String[])   - 
Method in class util.apps.gump. Gump 
  
   setMessage(String)   - 
Method in class bioroot. ContactBean 
  
   setMessages(String)   - 
Method in class bioroot. CellTypeBean 
  
   setMessages(String)   - 
Method in class bioroot. ContactBean 
  
   setMessages(String)   - 
Method in class bioroot. GeneBean 
  
   setMessages(String)   - 
Method in class bioroot. LoginBean 
  
   setMessages(String)   - 
Method in class bioroot. MarkerBean 
  
   setMessages(String)   - 
Method in class bioroot. OrganismBean 
  
   setMessages(String)   - 
Method in class bioroot. ReagentBean 
  
   setMessages(String)   - 
Method in class bioroot. SearchBean 
  
   setMessages(String)   - 
Method in class bioroot. UserRegistrationBean 
  
   setMessages(String)   - 
Method in class cgar.beans. LoginBean 
  
   setMiddle(int)   - 
Method in class trans.roc. RocWindow 
  
   setMinBpSides(int)   - 
Method in class trans.main. PeakPicker 
  
   setMinimum(double)   - 
Method in class trans.qc. StatFlag 
  
   setMinimum(double)   - 
Method in class util.gen. Histogram 
  
   setMinLength(int)   - 
Method in class util.bio.parsers. MultiFastaParser 
  
   setMinMaxY(double[])   - 
Method in class trans.graphics. Graph 
 Use to set a common y scale, pass it a minMax for the entire dataset.
   setMinPercent(double)   - 
Method in class trans.main. PeakPicker 
  
   setMinRun(int)   - 
Method in class trans.main. PeakPicker 
  
   setMinScore(double)   - 
Method in class gata.plotter. AlignPanel 
  
   setMinWindowFilter(float)   - 
Method in class edu.utah.seq.data. HeatMapMakerPosNeg 
  
   setMinWindowFilter(double)   - 
Method in class trans.main. WindowBlockMakerTwoColor 
  
   setMisc(double)   - 
Method in class trans.main. ScoredGene 
  
   setMls(int)   - 
Method in class util.apps. HouseBean 
  
   setModelValuesForScaling(double[])   - 
Method in class trans.cel. QuantileNormalization 
  
   setModifications(String)   - 
Method in class bioroot.oligo. OligoBean 
  
   setMotifDesc(String)   - 
Method in class meme. MemeMotif 
  
   setMotifHitPlotText(String)   - 
Method in class meme. MemeResults 
  
   setMotifHitsSummaryText(String)   - 
Method in class meme. MemeResults 
  
   setMotifResults(ArrayList)   - 
Method in class meme. MemeResults 
  
   setMotifSig(double)   - 
Method in class meme. MotifSearchResult 
  
   setMotifSites(String)   - 
Method in class meme. MemeMotif 
  
   setMotifSumLn(String)   - 
Method in class meme. MemeMotif 
  
   setMotifSummaryText(String)   - 
Method in class meme. MemeResults 
  
   setMutagen(String)   - 
Method in class bioroot.strain. StrainBean 
  
   setN(int)   - 
Method in class util.bio.parsers. BaseCount 
  
   setName(String)   - 
Method in class bioroot.antibody. AntibodyPrep 
  
   setName(String)   - 
Method in class bioroot. CellTypeBean 
  
   setName(String)   - 
Method in class bioroot. Feature 
  
   setName(String)   - 
Method in class bioroot. GeneBean 
  
   setName(String)   - 
Method in class bioroot. MarkerBean 
  
   setName(String)   - 
Method in class bioroot. OrganismBean 
  
   setName(String)   - 
Method in class bioroot. Organization 
  
   setName(String)   - 
Method in class bioroot.plasmid. OligoMatch 
  
   setName(String)   - 
Method in class bioroot. ReagentBean 
  
   setName(String)   - 
Method in class bioroot. TableHeading 
  
   setName(String)   - 
Method in class cgar.beans. AnalysisBean 
  
   setName(String)   - 
Method in class cgar.beans. AnalysisGroupBean 
  
   setName(String)   - 
Method in class cgar.beans. AnalysisTypeBean 
  
   setName(String)   - 
Method in class cgar.beans. GenomeBean 
  
   setName(String)   - 
Method in class cgar.beans. LabGroupBean 
  
   setName(String)   - 
Method in class cgar.beans. ProcessedDataBean 
  
   setName(String)   - 
Method in class cgar.beans. ProcessedDataTypeBean 
  
   setName(String)   - 
Method in class cgar.beans. RawDataBean 
  
   setName(int)   - 
Method in class cgar.beans. SpeciesBean 
  
   setName(String)   - 
Method in class cgar.beans. UserTypeBean 
  
   setName(String)   - 
Method in class cgar.beans. VisibilityBean 
  
   setName(String)   - 
Method in class edu.utah.seq.data. Info 
  
   setName(String)   - 
Method in class edu.utah.seq.methylation454. Reference 
  
   setName(String)   - 
Method in class trans.main. PosRegion 
  
   setName(String)   - 
Method in class trans.qc. StatFlag 
  
   setName(String)   - 
Method in class util.bio.cluster. Cluster 
  
   setName(String)   - 
Method in class util.bio.parsers. Fasta 
  
   setName(String)   - 
Method in class util.bio.parsers.gff. Gff3Feature 
  
   setName(String)   - 
Method in class util.bio.parsers. UCSCGeneLine 
  
   setName(String)   - 
Method in class util.gen. FileSize 
  
   setNAME_COMPSEQ(String)   - 
Method in class gata.aligner. AlignParams 
  
   setNAME_REFSEQ(String)   - 
Method in class gata.aligner. AlignParams 
  
   setNameChanged(boolean)   - 
Method in class bioroot.antibody. AntibodyBean 
  
   setNameLabel(JLabel)   - 
Method in class util.bio.cluster. Cluster 
  
   setNameLabelCenterYCoordinate(double)   - 
Method in class util.bio.cluster. Cluster 
  
   setNames(String[])   - 
Method in class trans.cel. ControlStats 
  
   setNamesAL(ArrayList)   - 
Method in class util.bio.parsers. MultiFastaParser 
  
   setNeighborhood(int)   - 
Method in class trans.anno. BindingRegion 
  
   setNgUl(double)   - 
Method in class bioroot.oligo. OligoBean 
  
   setNormalizedControlReplicas(File[])   - 
Method in class trans.main. T2Parameter 
  
   setNormalizedTreatmentReplicas(File[])   - 
Method in class trans.main. T2Parameter 
  
   setNote(String)   - 
Method in class util.bio.parsers.gff. Gff3Feature 
  
   setNotes(String)   - 
Method in class bioroot.antibody. AntibodyPrep 
  
   setNotes(String)   - 
Method in class bioroot.antibody. UseDilution 
  
   setNotes(String)   - 
Method in class bioroot. CellTypeBean 
  
   setNotes(String)   - 
Method in class bioroot. GeneBean 
  
   setNotes(String)   - 
Method in class bioroot. MarkerBean 
  
   setNotes(String)   - 
Method in class bioroot.oligo. OligoUploadBean 
  
   setNotes(String)   - 
Method in class bioroot. OrganismBean 
  
   setNotes(String)   - 
Method in class bioroot.plasmid. PlasmidUploadBean 
  
   setNotes(String)   - 
Method in class bioroot. ReagentBean 
  
   setNotes(String)   - 
Method in class bioroot.strain. StrainUploadBean 
  
   setNotes(String)   - 
Method in class cgar.beans. GenomeBean 
  
   setNotes(HashMap<String, String>)   - 
Method in class edu.utah.seq.data. Info 
  
   setNotes(String)   - 
Method in class trans.anno. Region 
  
   setNotes(StringBuffer)   - 
Method in class trans.qc. CelFileStats 
  
   setNotes(String)   - 
Method in class trans.roc. ControlGene 
  
   setNotes(String)   - 
Method in class util.apps.gump. Option 
  
   setNotes(String)   - 
Method in class util.bio.parsers. UCSCGeneLine 
  
   setNtAtXZero(double)   - 
Method in class gata.main. AnnoSpecParams 
  
   setNtCompSeq(int)   - 
Method in class gata.plotter. ToolsFrame 
  
   setNtPerPixel(double)   - 
Method in class gata.main. GATAParams 
  
   setNtRefSeq(int)   - 
Method in class gata.plotter. ToolsFrame 
  
   setNumber(int)   - 
Method in class util.bio.parsers. Fasta 
  
   setNumberControlIntensities(int)   - 
Method in class trans.main. Interval 
  
   SetNumberIntervalMaker   - Class in  trans.main  Determines the threshold(s) needed to generate a set number of intervals, multiple score indexes accepted.   SetNumberIntervalMaker(String[])   - 
Constructor for class trans.main. SetNumberIntervalMaker 
  
   setNumberIntervalMaker()   - 
Method in class trans.main. T2 
 Creates set number of intervals.
   setNumberMotifHits(int)   - 
Method in class trans.main. Interval 
  
   setNumberName(int)   - 
Method in class meme. MemeMotif 
  
   setNumberOfBins(int)   - 
Method in class util.gen. Histogram 
  
   setNumberOfWindows(int)   - 
Method in class edu.utah.seq.data. EnrichedRegion 
  
   setNumberOfWindows(int)   - 
Method in class trans.main. Interval 
  
   setNumberOligos(long)   - 
Method in class trans.main. ChromSet 
  
   setNumberPrepUseLines(int)   - 
Method in class bioroot.antibody. AntibodyBean 
  
   setNumberToDisplay(int)   - 
Method in class bioroot. Preference 
  
   setNumberTreatmentIntensities(int)   - 
Method in class trans.main. Interval 
  
   setNumBrightOutliers(int)   - 
Method in class trans.qc. CelFileStats 
  
   setNumDimOutliers(int)   - 
Method in class trans.qc. CelFileStats 
  
   setNumHitsAddOneLLPSPM(int)   - 
Method in class meme. MotifSearchResult 
  
   setNumHitsMemeLLPSPM(int)   - 
Method in class meme. MotifSearchResult 
  
   setNumNegatives(int)   - 
Method in class trans.roc. Bin 
  
   setNumNoSynthOutliers(int)   - 
Method in class trans.qc. CelFileStats 
  
   setNumOligos(int)   - 
Method in class trans.main. PeakPicker 
  
   setNumPositives(int)   - 
Method in class trans.roc. Bin 
  
   setObjectsFile(File)   - 
Method in class gata.main. GATAParams 
  
   setOk(boolean)   - 
Method in class trans.qc. CelFileStats 
  
   setOldFileName(String)   - 
Method in class bioroot.antibody. AntibodyBean 
  
   setOldFileName(String)   - 
Method in class bioroot.antibody. AntibodyPrep 
  
   setOligoBeans(OligoBean[])   - 
Method in class bioroot.oligo. OligoUploadBean 
  
   setOligoMatches(OligoMatch[])   - 
Method in class bioroot.plasmid. PlasmidBean 
  
   setOligoPreference(Preference)   - 
Method in class bioroot. UserBean 
  
   setOligoReports(String)   - 
Method in class bioroot.oligo. OligoUploadBean 
  
   setOligos(Oligo[])   - 
Method in class trans.main. Interval 
  
   setOligos(Oligo[])   - 
Method in class trans.main. PeakPicker 
  
   setOligos(Oligo[])   - 
Method in class trans.main. SubWindow 
  
   setOntologyTerm(String)   - 
Method in class util.bio.parsers.gff. Gff3Feature 
  
   setOptions(Option[])   - 
Method in class util.apps.gump. Gump 
  
   setOrder(double)   - 
Method in class bioroot. Feature 
  
   setOrderBy(String)   - 
Method in class bioroot. Preference 
  
   setOrderBy(String)   - 
Method in class bioroot. ReagentBean 
  
   setOrdered(boolean)   - 
Method in class bioroot. Preference 
  
   setOrderNumber(double)   - 
Method in class bioroot. TableHeading 
  
   setOrganismId(int)   - 
Method in class bioroot.antibody. AntibodyPrep 
  
   setOrganismId(int)   - 
Method in class bioroot. ReagentBean 
  
   setOrganismName(String)   - 
Method in class bioroot.antibody. AntibodyPrep 
  
   setOrganismName(String)   - 
Method in class bioroot. ReagentBean 
  
   setOrientation(String)   - 
Method in class bioroot.oligo. OligoBean 
  
   setOrientation(int)   - 
Method in class util.bio.annotation. GeneGroup 
  
   setOrientation(int)   - 
Method in class util.bio.wrappers. BL2SeqHit 
  
   setOverlapping(ArrayList)   - 
Method in class trans.main. PosRegion 
  
   setOwnerId(int)   - 
Method in class bioroot. ReagentBean 
  
   setOwnerName(String)   - 
Method in class bioroot. ContactBean 
  
   setOwnerName(String)   - 
Method in class bioroot. ReagentBean 
  
   setParent(String)   - 
Method in class util.bio.parsers.gff. Gff3Feature 
  
   setParentCluster(Cluster)   - 
Method in class util.bio.cluster. Cluster 
  
   setParentConcats(String[])   - 
Method in class bioroot.strain. StrainBean 
  
   setParentPoint(Point2D)   - 
Method in class util.bio.cluster. Cluster 
  
   setPassageNumber(String)   - 
Method in class bioroot.strain. StrainBean 
  
   setPassword(String)   - 
Method in class bioroot. LoginBean 
  
   setPassword(String)   - 
Method in class bioroot. UserBean 
  
   setPassword(String)   - 
Method in class cgar.beans. LoginBean 
  
   setPassword1(String)   - 
Method in class bioroot. UserRegistrationBean 
  
   setPassword2(String)   - 
Method in class bioroot. UserRegistrationBean 
  
   setPasswordReset(boolean)   - 
Method in class bioroot. UserRegistrationBean 
  
   setPeakBp(int)   - 
Method in class trans.main. PeakPicker 
  
   setPeakIndex(int)   - 
Method in class trans.main. PeakPicker 
  
   setPercentFinishedBasement(int)   - 
Method in class util.apps. HouseBean 
  
   setPermMeanDiff(float[][])   - 
Method in class trans.main. SamChromosomeData 
  
   setPermSamScores(float[], int, int)   - 
Method in class trans.main. SamChromosomeData 
  
   setPermTStats(float[][])   - 
Method in class trans.main. SamChromosomeData 
  
   setPermVariance(float[][])   - 
Method in class trans.main. SamChromosomeData 
  
   setPhase(int)   - 
Method in class util.bio.parsers.gff. Gff3Feature 
  
   setPhaseSet(boolean)   - 
Method in class util.bio.parsers.gff. Gff3Feature 
  
   setPhenotype(String)   - 
Method in class bioroot.strain. StrainBean 
  
   setPhone(String)   - 
Method in class bioroot. UserBean 
  
   setPhone(String)   - 
Method in class bioroot. UserRegistrationBean 
  
   setPixBtwDNATracks(double)   - 
Method in class gata.main. GATAParams 
  
   setPixBtwLabelTrack(double)   - 
Method in class gata.main. GATAParams 
  
   setPixBtwProtRNA(double)   - 
Method in class gata.main. GATAParams 
  
   setPixBtwTracks(double)   - 
Method in class gata.main. GATAParams 
  
   setPixBtwTransGrpLabel(double)   - 
Method in class gata.main. GATAParams 
  
   setPixBtwTransGrps(double)   - 
Method in class gata.main. GATAParams 
  
   setPixBtwTransGrpsGene(double)   - 
Method in class gata.main. GATAParams 
  
   setPixFatLineThickness(float)   - 
Method in class gata.main. GATAParams 
  
   setPixHeightArrows(double)   - 
Method in class gata.main. GATAParams 
  
   setPixNormalLineThickness(float)   - 
Method in class gata.main. GATAParams 
  
   setPixPerNt(double)   - 
Method in class gata.main. GATAParams 
  
   setPixThinLineThickness(float)   - 
Method in class gata.main. GATAParams 
  
   setPlasmidBeans(PlasmidBean[])   - 
Method in class bioroot.plasmid. PlasmidUploadBean 
  
   setPlasmidConcats(String[])   - 
Method in class bioroot.antibody. AntibodyBean 
  
   setPlasmidConcats(String[])   - 
Method in class bioroot.strain. StrainBean 
  
   setPlasmidPreference(Preference)   - 
Method in class bioroot. UserBean 
  
   setPlasmidReports(String)   - 
Method in class bioroot.plasmid. PlasmidUploadBean 
  
   setPlotterDefaults(GATAParams)   - 
Method in class gata.main. PlotterPreferences 
 Contains all the default preferences for GATAParams, alter these to change the default view
   setPlotterPreferences(PlotterPreferences)   - 
Method in class gata.main. GATAParams 
  
   setPngResultFile(File)   - 
Method in class util.bio.cluster. HierarchicalClustering 
  
   setPosition(int)   - 
Method in class edu.utah.seq.data. Point 
  
   setPosition(int)   - 
Method in class edu.utah.seq.parsers. ElandExportLine 
  
   setPosition(int)   - 
Method in class trans.roc. Gr 
  
   setPosition(int)   - 
Method in class util.bio.parsers. BaseCount 
  
   setPositions(int[])   - 
Method in class edu.utah.seq.data. PointData 
  
   setPreferences(String, String, String, String, String, Integer, Integer, Integer, Integer, String, String, String, String, String, String)   - 
Method in class gata.aligner. AlignerPreferences 
  
   setPresenceOfGFFs()   - 
Method in class gata.plotter. AlignPanel 
  
   setPrice(int)   - 
Method in class util.apps. HouseBean 
  
   setPrintMe(boolean)   - 
Method in class util.bio.xamer. Xamer 
  
   setPrintNotes(boolean)   - 
Method in class util.bio.parsers.gff. Gff3Parser 
  
   setProcessedDataTypeId(int)   - 
Method in class cgar.beans. ProcessedDataBean 
  
   setProgMon(String, int, ProgressMonitor)   - 
Method in class gata.aligner. AlignerThread 
  
   setProteinColor(Color)   - 
Method in class gata.main. GATAParams 
  
   setProteinVis(boolean)   - 
Method in class gata.main. GATAParams 
  
   setProtocolId(int)   - 
Method in class cgar.beans. AnalysisBean 
  
   setProtocolNotes(int)   - 
Method in class cgar.beans. AnalysisBean 
  
   setPurification(String)   - 
Method in class bioroot.antibody. AntibodyPrep 
  
   setPurification(String)   - 
Method in class bioroot.oligo. OligoBean 
  
   setPurification(String)   - 
Method in class bioroot.plasmid. PlasmidBean 
  
   setPvalue(float)   - 
Method in class expr. CorrelationWindow 
  
   setQuantiles(Quantile[][])   - 
Method in class trans.cel. QuantileNormalization 
  
   setRank(int)   - 
Method in class trans.graphics. GenomicRegion 
  
   setRank(float)   - 
Method in class trans.main. WilcoxonSamplePair 
  
   setRawMedian(double)   - 
Method in class trans.qc. CelFileStats 
  
   setRawScore(int)   - 
Method in class util.bio.wrappers. BL2SeqHit 
  
   setRawScoreCutOff(double)   - 
Method in class util.bio.wrappers. BL2Seq 
  
   setRawUnparsedResults(String[])   - 
Method in class util.bio.wrappers. BL2Seq 
  
   setReadLength(int)   - 
Method in class edu.utah.seq.data. Info 
  
   setReads(BaseRead[])   - 
Method in class edu.utah.seq.methylation454. CpG 
  
   setReagentNames(String)   - 
Method in class bioroot. ContactBean 
  
   setRealMeanDiff(float[])   - 
Method in class trans.main. SamChromosomeData 
  
   setRealSamScores(float[], int)   - 
Method in class trans.main. SamChromosomeData 
  
   setRealTStats(float[])   - 
Method in class trans.main. SamChromosomeData 
  
   setRealVariance(float[])   - 
Method in class trans.main. SamChromosomeData 
  
   setRecipients(String)   - 
Method in class bioroot.antibody. AntibodyPrep 
  
   setRecipients(String)   - 
Method in class bioroot.plasmid. PlasmidBean 
  
   setRecipients(String)   - 
Method in class bioroot.strain. StrainBean 
  
   setRefAnnoSpecParams(AnnoSpecParams)   - 
Method in class gata.main. GATAParams 
  
   setRefAnnotation(Annotation)   - 
Method in class gata.menu. GATAFrame 
 needed to add later since annotation doesn't exist when ToolFrame is created
   setReference(String)   - 
Method in class bioroot. ReagentBean 
  
   setReferenceY(double)   - 
Method in class gata.main. AnnoSpecParams 
  
   setRefGlyphPanel(GlyphPanel)   - 
Method in class gata.menu. GATAFrame 
  
   setRefs(JTextField, JFrame, JFileChooser)   - 
Method in class gata.aligner. FileFocusListener 
  
   setRegExGeneTypes(String)   - 
Method in class util.bio.parsers.gff. DmelRel4Extractor 
  
   setRegExTypes(String)   - 
Method in class util.bio.parsers.gff. Gff3Parser 
 Use something like -> gene|ncRNA|snoRNA|tRNA|rRNA|transposable_element|pseudogene|CDS|mRNA|exon 
 Only those gff lines with a type matching this regular expression will be made into GFF3Feature's.
   setRegion(PosRegion)   - 
Method in class trans.main. PosRegion 
  
   setRegionNames(String[])   - 
Method in class trans.main. Interval 
  
   setRelax(boolean)   - 
Method in class util.bio.parsers.gff. Gff3Parser 
  
   setRemainingAliquots(String)   - 
Method in class bioroot.antibody. AntibodyPrep 
  
   setRemainingAliquots(String)   - 
Method in class bioroot.strain. StrainBean 
  
   setRemoteHost(String)   - 
Method in class bioroot. UserBean 
  
   setRequesterEmail(String)   - 
Method in class bioroot. ContactBean 
  
   setResponse(String)   - 
Method in class bioroot. LoginBean 
  
   setRestock(String)   - 
Method in class bioroot.strain. StrainBean 
  
   setResultsDirectory(String)   - 
Method in class meme. MemeResults 
  
   setResultSummaryLine(String)   - 
Method in class util.bio.wrappers. Primer3Pick 
  
   setRevCompRef(Xamer)   - 
Method in class util.bio.xamer. Xamer 
  
   setRevCompRefs(ArrayList)   - 
Method in class util.bio.xamer. XamerCounter 
 Set refs to reverse comp xamer
   setRfa(RankedFloatArray)   - 
Method in class expr. ExpressedGene 
  
   setRightIndex(int)   - 
Method in class trans.main. PeakPicker 
  
   setRightSlope(double)   - 
Method in class trans.main. Oligo 
  
   setRNAColor(Color)   - 
Method in class gata.main. GATAParams 
  
   setRNAVis(boolean)   - 
Method in class gata.main. GATAParams 
  
   setS3Service(S3Service)   - 
Method in class util.amazon. S3Manager 
  
   setSaveDirectory(File)   - 
Method in class trans.main. ChromSet 
  
   setSavePNG(boolean)   - 
Method in class util.bio.cluster. HierarchicalClustering 
  
   setScaleBar(ScaleBar)   - 
Method in class gata.main. AnnoSpecParams 
  
   setScaleBarVis(boolean)   - 
Method in class gata.main. AnnoSpecParams 
  
   setScaleBarX(double)   - 
Method in class gata.main. AnnoSpecParams 
  
   setScaleBarY(double)   - 
Method in class gata.main. AnnoSpecParams 
  
   setScaleByControlStats(boolean)   - 
Method in class trans.cel. QuantileNormalization 
  
   setScaleLineColor(Color)   - 
Method in class gata.main. AnnoSpecParams 
  
   setScaleRulerVis(boolean)   - 
Method in class gata.main. AnnoSpecParams 
  
   setScaleRulerY(double)   - 
Method in class gata.main. AnnoSpecParams 
  
   setScore(float)   - 
Method in class edu.utah.seq.data. Point 
  
   setScore(float)   - 
Method in class edu.utah.seq.parsers. ElandExportLine 
  
   setScore(double)   - 
Method in class meme. MotifHit 
  
   setScore(float)   - 
Method in class trans.roc. Gr 
  
   setScore(double)   - 
Method in class trans.roc. RocWindow 
  
   setScore(double)   - 
Method in class trans.roc. Sgr 
  
   setScore(double)   - 
Method in class util.bio.annotation. ScoredCoordinate 
  
   setScore(double)   - 
Method in class util.bio.parsers.gff. Gff3Feature 
  
   setScore(String)   - 
Method in class util.bio.wrappers. BlastResult 
  
   setScoreAndSort(Window[])   - 
Method in class trans.main. FDRWindowConverter 
  
   setScoreCutOff(double)   - 
Method in class trans.main. PeakPicker 
  
   setScoreIndex(int)   - 
Method in class edu.utah.seq.data. HeatMapMaker 
  
   setScoreIndex(int)   - 
Method in class trans.main. WindowBlockMaker 
  
   setScores(float[])   - 
Method in class edu.utah.seq.data. PointData 
  
   setScores(float[])   - 
Method in class edu.utah.seq.data. SmoothingWindow 
  
   setScores(double, boolean)   - 
Method in class gata.plotter. ToolsFrame 
  
   setScores(double[])   - 
Method in class trans.main. Window 
  
   setScores(ArrayList)   - 
Method in class trans.roc. Positive 
  
   setScores()   - 
Method in class util.apps. GeneCentricCNVParser 
  
   setScores(float[])   - 
Method in class util.bio.parsers. UCSCGeneLine 
  
   setScoreSet(boolean)   - 
Method in class util.bio.parsers.gff. Gff3Feature 
  
   setScrollPaneScrollBarIncrement(JScrollPane, int)   - 
Static method in class gata.main. GATAUtil 
 Sets the scroll bar move increments, vert and horiz, in a scroll pane
   setSearchFor(String)   - 
Method in class bioroot. SearchBean 
  
   setSeedType(String)   - 
Method in class bioroot.strain. StrainBean 
  
   setSelexOligos(ArrayList)   - 
Method in class util.bio.xamer. Xamer 
  
   setSenseStrand(boolean)   - 
Method in class trans.roc. Positive 
  
   setSeq(String)   - 
Method in class meme. MotifHit 
  
   setSeq(String)   - 
Method in class util.bio.parsers. Fasta 
  
   setSeq1(String)   - 
Method in class util.bio.wrappers. BL2SeqHit 
  
   setSeq2(String)   - 
Method in class util.bio.wrappers. BL2SeqHit 
  
   setSeqId(String)   - 
Method in class util.bio.parsers.gff. Gff3Feature 
  
   setSeqs(String, String)   - 
Method in class gata.aligner. Alignment 
  
   setSeqsAL(ArrayList)   - 
Method in class util.bio.parsers. MultiFastaParser 
  
   setSequence(String)   - 
Method in class bioroot.oligo. OligoBean 
  
   setSequence(String)   - 
Method in class bioroot.plasmid. PlasmidBean 
  
   setSequence(String)   - 
Method in class edu.utah.seq.data. EnrichedRegion 
  
   setSequence(String)   - 
Method in class edu.utah.seq.methylation454. Read 
  
   setSequence(String)   - 
Method in class trans.main. Interval 
  
   setSequence(String)   - 
Method in class trans.main. Oligo 
  
   setSequence(String)   - 
Method in class trans.tpmap. TPMapLine 
  
   setSequence(String)   - 
Method in class util.bio.seq. IndexedSequence 
  
   setSequence(String)   - 
Method in class util.bio.wrappers. Primer3Pick 
  
   setSimpleMulti(S3ServiceSimpleMulti)   - 
Method in class util.amazon. S3Manager 
  
   setSize(int)   - 
Method in class util.apps. HouseBean 
  
   setSize(long)   - 
Method in class util.gen. FileSize 
  
   setSizeOfOligoMinusOne(int)   - 
Method in class trans.main. Interval 
  
   setSkip(boolean)   - 
Method in class trans.main. Oligo 
  
   setSm(SmoothingWindow[])   - 
Method in class edu.utah.seq.data. SmoothingWindowInfo 
  
   setSmallestAlignPanelY(int)   - 
Method in class gata.main. GATAParams 
  
   setSmallestGlyphPanelY(int)   - 
Method in class gata.main. AnnoSpecParams 
  
   setSmoothedScore(double)   - 
Method in class trans.main. Oligo 
  
   setSortBy(double)   - 
Method in class bioroot. BlastMatcher 
  
   setSortBy(double)   - 
Method in class trans.main. Interval 
  
   setSortBy(double)   - 
Method in class trans.main. Window 
  
   setSortBy(String)   - 
Method in class util.bio.parsers.gff. Gff3Feature 
  
   setSortByValue(boolean)   - 
Method in class trans.cel. ComparatorMultiSetQuantile 
  
   setSortByValue(boolean)   - 
Method in class trans.cel. ComparatorQuantile 
  
   setSource(String)   - 
Method in class bioroot.antibody. AntibodyBean 
  
   setSource(String)   - 
Method in class bioroot.plasmid. PlasmidBean 
  
   setSource(String)   - 
Method in class bioroot.strain. StrainBean 
  
   setSource(String)   - 
Method in class cgar.beans. GenomeBean 
  
   setSource(String)   - 
Method in class util.bio.parsers.gff. Gff3Feature 
  
   setSpeciesId(int)   - 
Method in class cgar.beans. GenomeBean 
  
   setSpinner(JSpinner, int, int)   - 
Method in class gata.aligner. AlignerInputPanel 
  
   setStart(int)   - 
Method in class edu.utah.seq.data. EnrichedRegion 
  
   setStart(int)   - 
Method in class edu.utah.seq.data. SmoothingWindow 
  
   setStart(int)   - 
Method in class meme. MotifHit 
  
   setStart(int)   - 
Method in class trans.anno. Region 
  
   setStart(int)   - 
Method in class trans.main. PosRegion 
  
   setStart(double)   - 
Method in class trans.roc. Bin 
  
   setStart(int)   - 
Method in class trans.roc. RocWindow 
  
   setStart(int)   - 
Method in class util.bio.annotation. Coordinate 
  
   setStart(int)   - 
Method in class util.bio.annotation. ExonIntron 
  
   setStart(int)   - 
Method in class util.bio.parsers.gff. Gff3Feature 
  
   setStart1stOligo(int)   - 
Method in class trans.main. Interval 
  
   setStart1stOligo(int)   - 
Method in class trans.main. Window 
  
   setSTART_INDEX_COMPSEQ(int)   - 
Method in class gata.aligner. AlignParams 
  
   setSTART_INDEX_REFSEQ(int)   - 
Method in class gata.aligner. AlignParams 
  
   setStartLastOligo(int)   - 
Method in class trans.main. Interval 
  
   setStartLastOligo(int)   - 
Method in class trans.main. Window 
  
   setStartPosition(int)   - 
Method in class edu.utah.seq.methylation454. Reference 
  
   setStartSeq1(int)   - 
Method in class util.bio.wrappers. BL2SeqHit 
  
   setStartSeq2(int)   - 
Method in class util.bio.wrappers. BL2SeqHit 
  
   setStartSet(boolean)   - 
Method in class util.bio.parsers.gff. Gff3Feature 
  
   setState(String)   - 
Method in class util.apps. HouseBean 
  
   setStats(double[])   - 
Method in class trans.cel. ControlStats 
  
   setStatus(String)   - 
Method in class util.apps. HouseBean 
  
   setStndDev(double)   - 
Method in class trans.qc. StatFlag 
  
   setStop(int)   - 
Method in class edu.utah.seq.data. EnrichedRegion 
  
   setStop(int)   - 
Method in class edu.utah.seq.data. SmoothingWindow 
  
   setStop(int)   - 
Method in class meme. MotifHit 
  
   setStop(double)   - 
Method in class trans.roc. Bin 
  
   setStop(int)   - 
Method in class util.bio.annotation. Coordinate 
  
   setStopSeq1(int)   - 
Method in class util.bio.wrappers. BL2SeqHit 
  
   setStopSeq2(int)   - 
Method in class util.bio.wrappers. BL2SeqHit 
  
   setStrainBeans(StrainBean[])   - 
Method in class bioroot.strain. StrainUploadBean 
  
   setStrainConcats(String[])   - 
Method in class bioroot.antibody. AntibodyBean 
  
   setStrainConcats(String[])   - 
Method in class bioroot.plasmid. PlasmidBean 
  
   setStrainPreference(StrainPreference)   - 
Method in class bioroot. UserBean 
  
   setStrainReports(String)   - 
Method in class bioroot.strain. StrainUploadBean 
  
   setStrainTypes(String[])   - 
Method in class bioroot.strain. StrainPreference 
  
   setStrand(String)   - 
Method in class edu.utah.seq.data. Info 
  
   setStrand(String)   - 
Method in class edu.utah.seq.methylation454. Read 
  
   setStrand(String)   - 
Method in class edu.utah.seq.parsers. BarParser 
  
   setStrand(String)   - 
Method in class util.bio.parsers.gff. Gff3Feature 
  
   setStrand(String)   - 
Method in class util.bio.wrappers. BlastResult 
  
   setStreetAddress(String)   - 
Method in class util.apps. HouseBean 
  
   setSubmitterUserBean(UserBean)   - 
Method in class bioroot. UserRegistrationBean 
  
   setSubWindows(SmoothingWindow[])   - 
Method in class edu.utah.seq.data. EnrichedRegion 
  
   setSubWindowScoreAsMinScore()   - 
Method in class trans.main. PeakPicker 
 Averages the smoothed scores in the best sub window.
   setT(int)   - 
Method in class util.bio.parsers. BaseCount 
  
   setTableName(String)   - 
Method in class bioroot. SearchBean 
  
   setTagValues(HashMap<String, String>)   - 
Method in class edu.utah.seq.parsers. BarParser 
  
   setTarget(String)   - 
Method in class util.bio.parsers.gff. Gff3Feature 
 Reserved Attribute.
   setTargetMedian(float)   - 
Method in class trans.cel. QuantileNormalization 
  
   setText(String)   - 
Method in class util.apps.gump. GumpResultsScrollPane 
  
   setTextArea(String)   - 
Method in class gata.plotter. Console 
  
   setTextArea(String)   - 
Method in class util.apps.gump. GumpResults 
  
   setThreadedResults(Map)   - 
Method in class util.amazon. S3Manager 
  
   setTitle(String)   - 
Method in class util.bio.cluster. HierarchicalClustering 
  
   setToolsFrameRef(ToolsFrame)   - 
Method in class gata.main. GATAParams 
  
   setToolsRef(ToolsFrame)   - 
Method in class gata.plotter. AlignPanel 
  
   setTopCluster(Cluster)   - 
Method in class util.bio.cluster. Cluster 
  
   setTopCuts(int[])   - 
Method in class util.bio.digest. Enzyme 
  
   setTotalClusters(double)   - 
Method in class util.bio.cluster. Cluster 
  
   setTotalNumberWindows(int)   - 
Method in class trans.roc. Positive 
  
   setTpmapInfo(ArrayList)   - 
Method in class trans.cel. QuantileNormalization 
  
   setTrackDefaults()   - 
Method in class gata.geneGlyphs. Annotation 
  
   setTrackedGenericGlyphs(GenericGlyph[][])   - 
Method in class gata.main. AnnoSpecParams 
  
   setTrackLabels(ArrayList[])   - 
Method in class gata.main. AnnoSpecParams 
  
   setTrackLabelVis(boolean[])   - 
Method in class gata.main. AnnoSpecParams 
  
   setTrackNumber(int)   - 
Method in class gata.main. AnnoSpecParams 
  
   setTrackNumber(int)   - 
Method in class util.bio.annotation. GenericFeature 
  
   setTrackParams()   - 
Method in class gata.geneGlyphs. GlyphPanel 
  
   setTrackThickness(float[])   - 
Method in class gata.main. AnnoSpecParams 
  
   setTrackVis(boolean[])   - 
Method in class gata.main. AnnoSpecParams 
  
   setTransGrp(TransGroup)   - 
Method in class util.bio.annotation. Translation 
 Sets TransGroup ref but also fires the makeSegments method
   setTransGrpGlyphs(ArrayList)   - 
Method in class gata.geneGlyphs. GeneRepGlyph 
  
   setTreatmentPoints(Point[])   - 
Method in class edu.utah.seq.data. EnrichedRegion 
  
   setTreatmentReplica(boolean)   - 
Method in class trans.main. ChromSet 
  
   setTss(int)   - 
Method in class util.bio.parsers. UCSCGeneLine 
  
   setTwoTailed(boolean)   - 
Method in class trans.main. WilcoxonRankSumTest 
  
   setTxEnd(int)   - 
Method in class util.bio.parsers. UCSCGeneLine 
  
   setTxStart(int)   - 
Method in class util.bio.parsers. UCSCGeneLine 
  
   setType(String)   - 
Method in class bioroot.antibody. AntibodyBean 
  
   setType(String)   - 
Method in class bioroot. ContactBean 
  
   setType(String)   - 
Method in class bioroot.oligo. OligoBean 
  
   setType(String)   - 
Method in class bioroot.strain. StrainBean 
  
   setType(String)   - 
Method in class bioroot. UserBean 
  
   setType(String)   - 
Method in class bioroot. UserRegistrationBean 
  
   setType(int)   - 
Method in class trans.roc. RocWindow 
  
   setType(String)   - 
Method in class util.bio.parsers.gff. Gff3Feature 
  
   setTypeId(int)   - 
Method in class cgar.beans. UserTypeBean 
  
   setUM(double)   - 
Method in class bioroot.oligo. OligoBean 
  
   setUri(String)   - 
Method in class cgar.beans. GenomeBean 
  
   setUri(String)   - 
Method in class cgar.beans. ProcessedDataBean 
  
   setUri(String)   - 
Method in class cgar.beans. RawDataBean 
  
   setUseDilutions(UseDilution[])   - 
Method in class bioroot.antibody. AntibodyPrep 
  
   setUseMMData(boolean)   - 
Method in class trans.cel. QuantileNormalization 
  
   setUserComments(String)   - 
Method in class bioroot.antibody. AntibodyPrep 
  
   setUserId(int)   - 
Method in class bioroot. UserRegistrationBean 
  
   setUserId(int)   - 
Method in class cgar.beans. AnalysisBean 
  
   setUserInput(String)   - 
Method in class util.apps.gump. Option 
  
   setUserInputS(JTextField)   - 
Method in class util.apps.gump. Option 
  
   setValid(boolean)   - 
Method in class util.bio.parsers.gff. Gff3Feature 
  
   setValues(float[])   - 
Method in class edu.utah.seq.parsers. BarParser 
  
   setValues(float[])   - 
Method in class expr. ExpressedGene 
  
   setValues(float[])   - 
Method in class trans.misc. GrGraph 
  
   setVersionedGenome(String)   - 
Method in class edu.utah.seq.data. Info 
  
   setVersionedGenome(String)   - 
Method in class edu.utah.seq.parsers. BarParser 
  
   setViewPort(JViewport)   - 
Method in class gata.geneGlyphs. GlyphPanel 
  
   setViewPort(JViewport)   - 
Method in class gata.plotter. AlignPanel 
  
   setVisibility(String)   - 
Method in class bioroot. Preference 
  
   setVisibility(String)   - 
Method in class bioroot. ReagentBean 
  
   setVisibilityId(int)   - 
Method in class cgar.beans. AnalysisBean 
  
   setVisible(boolean)   - 
Method in class bioroot. Feature 
  
   setVisible(boolean)   - 
Method in class bioroot. TableHeading 
  
   setVolume(String)   - 
Method in class bioroot.oligo. OligoBean 
  
   setVolume(String)   - 
Method in class bioroot.plasmid. PlasmidBean 
  
   setW(int)   - 
Method in class util.bio.wrappers. BL2Seq 
  
   setWidth(int)   - 
Method in class gata.main. GATAParams 
  
   setWidth(int)   - 
Method in class util.apps.gump. GumpDrawFrame 
  
   setWindowsVisibility(boolean)   - 
Method in class gata.menu. GATAFrame 
 Add new windows/panels here to enable them to be shown or hidden in sync with the main frame
   setWsps(WilcoxonSamplePair[])   - 
Method in class trans.main. WilcoxonSignedRankTest 
  
   setXamer(String)   - 
Method in class util.bio.xamer. Xamer 
  
   setYearBuilt(int)   - 
Method in class util.apps. HouseBean 
  
   setZipCode(int)   - 
Method in class util.apps. HouseBean 
  
   setZipCompress(boolean)   - 
Method in class edu.utah.seq.parsers. BarParser 
  
   setZoomedWidth(int)   - 
Method in class gata.main. GATAParams 
  
   Sgr   - Class in  trans.roc  Sgr line container.   Sgr(String)   - 
Constructor for class trans.roc. Sgr 
  
   Sgr(String, String)   - 
Constructor for class trans.roc. Sgr 
  
   Sgr2Bar   - Class in  trans.misc      Sgr2Bar(String[])   - 
Constructor for class trans.misc. Sgr2Bar 
  
   SgrBacCounter   - Class in  trans.roc  Application for scoring '.sgr' files for positives and negatives in relation to the BAC spike in data.   SgrBacCounter(String[])   - 
Constructor for class trans.roc. SgrBacCounter 
  
   sgrBundle()   - 
Method in class util.bio.parsers.gff. Gff3Feature 
 Returns a collection of sgr lines representing the gff feature:
 1) seqId startMin1 0
 2) seqId start score
 3) seqId end score
 4) seqId endPlus1 0
 Does not include a final return, thus println(gff.sgrBundle()).
   SgrComparator   - Class in  trans.misc      SgrComparator()   - 
Constructor for class trans.misc. SgrComparator 
  
   ShiftPositionsInUCSCGeneLines   - Class in  util.bio.parsers  Shifts the bp position of UCSCGeneLines matching particular chromosomes.Good for shifting coordinates after making a composite mock chromosome from 
 unassembled sequences/ scaffolds.   ShiftPositionsInUCSCGeneLines()   - 
Constructor for class util.bio.parsers. ShiftPositionsInUCSCGeneLines 
  
   showFileChooserDialogBox(JTextField, JFrame, JFileChooser)   - 
Static method in class gata.main. GATAUtil 
  
   showFileChooserDialogBox(JTextField, JFrame, JFileChooser)   - 
Static method in class util.gen. Swing 
  
   shuffleCards()   - 
Method in class util.apps. Flash 
 Randomly permutates the FlashCard[].
   shuffleIntensities()   - 
Method in class trans.main. ScanChip 
 Combines and shuffles the treatment and control intensities.
   shuffleIntensityLabels()   - 
Method in class trans.main. ScanChromosomes 
 Combines and shuffles the treatment and control intensities.
   shuffleIntensityLabels()   - 
Method in class trans.main. ScanChromosomesCNV 
 Combines and shuffles the treatment and control intensities.
   shuffleIntensityPositions()   - 
Method in class trans.main. ScanChromosomes 
 Shuffles the treatment and control intensities (seperately).
   shuffleIntensityPositions()   - 
Method in class trans.main. ScanChromosomesCNV 
 Shuffles the treatment and control intensities (seperately).
   signedDifference(float[], float)   - 
Static method in class trans.main. WilcoxonSignedRankTest 
 Returns signed difference between the scores and a median.
   SimpleBlast   - Class in  util.bio.wrappers  Wrapper for bl2seq blast.   SimpleBlast(String)   - 
Constructor for class util.bio.wrappers. SimpleBlast 
  
   simpleMulti   - 
Variable in class util.amazon. S3Manager 
  
   simpleSummaryLine()   - 
Method in class trans.anno. BindingRegion 
 Returns a binding region line, tab delimited: chrom, start, end, score
   simpleSummaryLine()   - 
Method in class trans.roc. RocWindow 
 Returns a binding region line, tab delimited: chrom, start, end, score
   simpleSummaryLineWithRank()   - 
Method in class trans.anno. BindingRegion 
 Returns a binding region line, tab delimited: chrom, start, end, score
   SimpleTextWindow   - Class in  gata.main      SimpleTextWindow(String, int, int, String)   - 
Constructor for class gata.main. SimpleTextWindow 
  
   simpleToString()   - 
Method in class util.bio.parsers. UCSCGeneLine 
  
   Simulator   - Class in  edu.utah.seq.data      Simulator(String[])   - 
Constructor for class edu.utah.seq.data. Simulator 
 Constructor
   skip()   - 
Method in class trans.main. Oligo 
  
   skipHeader(BufferedReader)   - 
Method in class util.bio.parsers. Cap3Parser 
  
   skipToFeatures(BufferedReader)   - 
Method in class util.bio.parsers. RefSeqAnnotationParser 
  
   sleep(int)   - 
Static method in class util.gen. Misc 
 Sleeps for given number of seconds
   Sliders   - Class in  gata.plotter      Sliders(AlignParams, GATAParams)   - 
Constructor for class gata.plotter. Sliders 
  
   slopeOligos()   - 
Method in class trans.main. PeakPicker 
  
   SMALLFONT   - 
Static variable in class util.apps.gump. GumpToolPanel 
  
   SmoothData   - Class in  util.apps  Pulls the first two columns of double from a white space delimited file.   SmoothData()   - 
Constructor for class util.apps. SmoothData 
  
   SmoothingWindow   - Class in  edu.utah.seq.data  Coordinates are interbase!   SmoothingWindow()   - 
Constructor for class edu.utah.seq.data. SmoothingWindow 
  
   SmoothingWindow(int, int, float[])   - 
Constructor for class edu.utah.seq.data. SmoothingWindow 
  
   SmoothingWindowInfo   - Class in  edu.utah.seq.data  Chromosome specific container for window level results from EnrichedRegionMaker.   SmoothingWindowInfo(SmoothingWindow[], Info)   - 
Constructor for class edu.utah.seq.data. SmoothingWindowInfo 
  
   smoothScores(double[], int[], int)   - 
Static method in class util.gen. Num 
 Uses a sliding window to smooth the scores applying a trimmed mean (drop 1 from ends) to the scores found within the window.
   SMTPAccount   - 
Static variable in class bioroot. Util 
 SMTP account to use for emailing
   smtpHostName   - 
Static variable in class bioroot. Util 
  
   smtpPassword   - 
Static variable in class bioroot. Util 
  
   smtpUser   - 
Static variable in class bioroot. Util 
  
   sortIntervals()   - 
Method in class trans.main. IntervalGraphPrinter 
 Sorts Interval[] based on subWindow if present, if not, then uses best window
   sortIntervalsByChromosome(Interval[])   - 
Static method in class trans.main. LoadIntervalOligoInfo 
 Ugly, ugly, ugly, use a Comparator!
   sortIntervalsBySubWindowMedianRatio(Interval[])   - 
Static method in class trans.misc. Util 
 Sorts an array of Interval by the median ratio of the best SubWindow.
   sortMotifSearchResults()   - 
Method in class meme. MemeResults 
 method to sort the array of MotifSearchResult objects, also sets two fields based on the this.fileNameHash
   sortQuantileArrays(Quantile[][])   - 
Method in class trans.cel. QuantileNormalization 
 Sorts quantile arrays based on comparator, either position or intensity value.
   sortSegments(ArrayList)   - 
Static method in class gata.main. GATAUtil 
 Sorts an ArrayList of int[2]'s representing start, end
   sortSegments(ArrayList)   - 
Static method in class util.bio.annotation. GeneGroup 
 Sorts an ArrayList of int[2]'s representing start, end
   SOURCE_TAG   - 
Static variable in class edu.utah.seq.parsers. BarParser 
  
   space   - 
Static variable in class util.apps.gump. Option 
  
   spaces(String, int)   - 
Static method in class gata.main. GATAUtil 
  
   Spammer   - Class in  bioroot  For sending out email messages   Spammer(String[])   - 
Constructor for class bioroot. Spammer 
  
   SpearmanCorrelation   - Class in  util.gen  Calculates a Spearman correlation coefficient (rho), in the case of ties, averages the ranks 
 and returns a Pearson correlation coefficient on the ranks.   SpearmanCorrelation()   - 
Constructor for class util.gen. SpearmanCorrelation 
  
   spearmanCorrelationCoefficient(float[], float[])   - 
Method in class util.gen. SpearmanCorrelation 
 Returns a Spearman Rank Correlation Coefficient (rho) between the 
 two arrays.
   spearmanCorrelationCoefficient(RankedFloatArray, RankedFloatArray)   - 
Method in class util.gen. SpearmanCorrelation 
 Returns a Spearman Rank Correlation Coefficient (rho) between the 
 two arrays.
   SpeciesBean   - Class in  cgar.beans  Name of a species from which multiple builds will be associated.   SpeciesBean()   - 
Constructor for class cgar.beans. SpeciesBean 
  
   splineNormalize()   - 
Method in class trans.cel. QuantileNormalization 
 Uses a cubic spline and user defined regions to non-linearly scale the data.
   SplineScalar   - Class in  trans.cel  Generates multiplicative scalars based on a set of points using a combination of a cubic spline for values within the provided points 
 and linear regression for points outside the value range.   SplineScalar(double[])   - 
Constructor for class trans.cel. SplineScalar 
 Assumes modelPoints are sorted min to max.
   split(HashMap<String, PointData>)   - 
Static method in class edu.utah.seq.data. PointData 
 Splits the data in half using random sampling.
   split(boolean[])   - 
Method in class edu.utah.seq.data. PointData 
 Splits this.PointData's positions and scores based on the boolean[].
   splitAndClean(String)   - 
Method in class util.bio.parsers. RefSeqAnnotationParser 
  
   splitAndSaveIntensities(IntensityFeature[], File)   - 
Static method in class trans.cel. MakeChromosomeSets 
 Saves the intensities to file as a int[]
   splitAndSavePositions(IntensityFeature[], File)   - 
Static method in class trans.cel. MakeChromosomeSets 
 Saves the positions to file as a int[]
   splitAntibodyFeatures(Feature[])   - 
Static method in class bioroot.antibody. AntibodyBase 
 Returns arrays of Features where the F[0] = antibody, F[1]=abPrep, F[2]=useDil 
 specific features.
   splitByChrom(Sgr[])   - 
Static method in class trans.roc. Sgr 
 Splits a Sgr[] by chromosome.
   splitByChromosome(File)   - 
Method in class edu.utah.seq.parsers. ElandSequenceParser 
  
   splitByChromosome(ExpressedGene[])   - 
Static method in class expr. ExpressedGene 
 Splits a ExpressedGene[] by chromosome to ExpressedGene[chrom][lines].
   splitByChromosome(Region[])   - 
Static method in class trans.anno. Region 
 Given a chromosome SORTED array of Regions returns a HashMap 
 containing chromosome: sorted chromo specific Region[]
   splitByChromosome(Sgr[])   - 
Static method in class trans.roc. Sgr 
 Splits a Sgr[] containing many chromosome lines to a Sgr[chrom][lines].
   splitByChromosome(Coordinate[])   - 
Static method in class util.bio.annotation. Coordinate 
 Split by chromosome into a HashMap of chromosome:Coordinate[].
   splitByChromosome(ScoredCoordinate[])   - 
Static method in class util.bio.annotation. ScoredCoordinate 
 Split by chromosome into a HashMap of chromosome:ScoredCoordinate[].
   splitByChromosome()   - 
Method in class util.bio.parsers. UCSCGeneModelTableReader 
 Splits a UCSCGeneLine[] by chromosome into a HashMap of chromosome:UCSCGeneLine[].
   splitChromSetArray()   - 
Method in class trans.main. T2 
 Pulls and places replica directories to this.
   splitConcat(String)   - 
Static method in class bioroot. Util 
 Safely splits a three item, colon delimited concat (ie 3: zyx:4-99: 235, 3999) into a String[3], 
 OK to have internal colons provided no space follows the colon.
   splitGffToTypes()   - 
Method in class util.bio.parsers.gff. ParseWormBaseGffToUCSC 
  
   splitIndex(String, String)   - 
Method in class util.bio.seq. IndexFastas 
  
   splitIntervalArrayListByChromosome(ArrayList)   - 
Static method in class trans.misc. Util 
 Splits an ArrayList containing Interval by chromosome into a HashMap.
   splitOnFirstColon(String)   - 
Static method in class util.gen. Misc 
 Returns a String[2] split on first colon, if no colon returns null.
   splitString(String, String)   - 
Static method in class util.gen. Misc 
 Splits a String on a regular expression, watching out for escaped characters
 ie Cat\=Mouse would not be split when = is given as the regex, a String[0], 
 {"Cat\=Mouse"} would be returned.
   splitTagToTemp()   - 
Method in class edu.utah.seq.parsers. Tag2Point 
 Splits a tag file by chromosome and strand to seperate files.
   splitTPMap()   - 
Method in class trans.tpmap. MapSplitter 
  
   SplitWindowArraysByChromosome   - Class in  trans.main      SplitWindowArraysByChromosome(String[])   - 
Constructor for class trans.main. SplitWindowArraysByChromosome 
  
   splitWindowsByChromosome(Window[])   - 
Static method in class trans.misc. Util 
  
   SQL   - Class in  util.gen  Utility class to hold database and relevant fields and methods, some from Hall and Brown,
 most from BioRoot.   SQL(String, String, String, String, String)   - 
Constructor for class util.gen. SQL 
  
   SQL()   - 
Constructor for class util.gen. SQL 
  
   SQUARE_ROOTS   - 
Static variable in class expr. CorrelationMaps 
  
   squareRoots(int)   - 
Static method in class util.gen. Num 
 Returns the integer square root from zero to num.
   standard(String[])   - 
Static method in class trans.misc. InefficientPseudoMedian 
  
   standardDeviation(int[])   - 
Static method in class util.gen. Num 
 Calculates standard deviation of an int[]
   standardDeviation(int[], double)   - 
Static method in class util.gen. Num 
 Calculates standard deviation of an int[] and mean.
   standardDeviation(float[])   - 
Static method in class util.gen. Num 
 Calculates standard deviation of an float[]
   standardDeviation(float[], double)   - 
Static method in class util.gen. Num 
 Calculates standard deviation of an float[] given a mean.
   standardDeviation(double[])   - 
Static method in class util.gen. Num 
 Calculates standard deviation of an double[]
   standardDeviation(double[], double)   - 
Static method in class util.gen. Num 
 Calculates standard deviation of a double[] and mean.
   standardDeviationOfDifference(int[], int[])   - 
Static method in class util.gen. Num 
 Calculates the standard deviation of the difference between two int[]s 
 of the same length.
   standardError(double[], double)   - 
Static method in class util.gen. Num 
 Calculates standard error of a double[] given it's mean.
   start   - 
Variable in class trans.tpmap. IntensityFeature 
  
   start   - 
Variable in class trans.tpmap. MapFeature 
  
   startsInBases(StartStop[])   - 
Static method in class util.bio.annotation. StartStop 
 Returns the starts in number of bases, not coordinates for the array.
   startSpace   - 
Static variable in class util.apps.gump. Gump 
  
   StartStop   - Class in  gata.plotter      StartStop(int, int)   - 
Constructor for class gata.plotter. StartStop 
  
   StartStop   - Class in  util.bio.annotation  Simple start end object for sorting.   StartStop(int, int)   - 
Constructor for class util.bio.annotation. StartStop 
  
   StartStop(int[])   - 
Constructor for class util.bio.annotation. StartStop 
  
   Stat1lq   - Class in  trans.tpmap      Stat1lq()   - 
Constructor for class trans.tpmap. Stat1lq 
  
   StatBarFiles   - Class in  util.apps      StatBarFiles()   - 
Constructor for class util.apps. StatBarFiles 
  
   statBindingRegionArray(BindingRegion[])   - 
Static method in class trans.anno. IntersectRegions 
  
   statBindingRegionArray(BindingRegion[])   - 
Static method in class util.bio.parsers. FetchGenomicSequences 
  
   statCpGs(Sample, Sample)   - 
Method in class edu.utah.seq.methylation454. StatMethylationData 
 Generates two tailed Chi-square p-values for differences in %C (a proxy for %methylation) for each CpG in each Amplicon between two samples
 and a Wilcoxon Signed Rank p-value for all of the CpG %Cs in an amplicon.
   StatFlag   - Class in  trans.qc  Info related to a particular statistic used to measure a cel file.   StatFlag(String, double, double)   - 
Constructor for class trans.qc. StatFlag 
  
   statFloatArray(float[], boolean)   - 
Static method in class util.gen. Num 
 Calculates Min, Max, Mean, Median, Mode, and Histogram/10 for a Float[]
   StatIntensityFeatureArray   - Class in  trans.cel      StatIntensityFeatureArray()   - 
Constructor for class trans.cel. StatIntensityFeatureArray 
  
   StatMethylationData   - Class in  edu.utah.seq.methylation454      StatMethylationData(String[])   - 
Constructor for class edu.utah.seq.methylation454. StatMethylationData 
  
   statSingleCelFile(CelFileStats)   - 
Method in class trans.qc. CelFileQualityControl 
 Checks a CelStats object for deviations from the min max settings.
   StatTPMap   - Class in  trans.tpmap  Calculates simple stats on a tpmap file, number unique oligos, number of lines, gc, tm...   StatTPMap()   - 
Constructor for class trans.tpmap. StatTPMap 
  
   StatTPMapControlSeqs   - Class in  trans.tpmap      StatTPMapControlSeqs()   - 
Constructor for class trans.tpmap. StatTPMapControlSeqs 
  
   StrainBase   - Class in  bioroot.strain      StrainBase()   - 
Constructor for class bioroot.strain. StrainBase 
  
   StrainBean   - Class in  bioroot.strain      StrainBean()   - 
Constructor for class bioroot.strain. StrainBean 
  
   StrainBean(int, DBUtil)   - 
Constructor for class bioroot.strain. StrainBean 
  
   StrainBean(ResultSet)   - 
Constructor for class bioroot.strain. StrainBean 
  
   StrainBean(String[], IdNameMatch[], IdNameMatch[], IdNameMatch[], UserBean, DBUtil)   - 
Constructor for class bioroot.strain. StrainBean 
 For bulk file upload should be 23 cells 
0 Name
1 Alias
2 Source
4 Reference
5 Type Bacteria, Yeast, Worm, Insect, Plant, Fish, Cell Line, Bird, Mammal or Other
6 Recipients
7 Organism name
8 Markers names, comma seperated
9 Plasmid Ids
10 Parent Strain Ids
11 Storage Location
12 Mutagen   EMS, ENU, MMS, MNU, X-ray, UV, Transposon, T-DNA, Spontaneous or Other
13 Genotype/ Background
14 Phenotype
15 Growth Conditions
16 Restock
17 Remaining Aliquots
18 Array Type   Extra Chromosomal or Integration
19 Seed Type     Individual Line, Individual Pool, Set of Lines, or Set of Pools
20 Cell Type (Cell Lines)
21 Passage Number ( Cell Lines)
22 Mating Type ( Yeast) diploid, a, alpha, h+, or h-)
23 Availability
24 VisibilityBean
25 Notes
   StrainModify   - Class in  bioroot.strain  Servlet to modify strains using StrainBeans referenced from a Session object.   StrainModify()   - 
Constructor for class bioroot.strain. StrainModify 
  
   StrainPreference   - Class in  bioroot.strain      StrainPreference()   - 
Constructor for class bioroot.strain. StrainPreference 
  
   StrainPreference(String)   - 
Constructor for class bioroot.strain. StrainPreference 
 databaseConcat = visibility | orderBy| numberToDisplay | labGroups | features | strain types   String[] separated by semi-colons
   StrainSpreadSheet   - Class in  bioroot.strain      StrainSpreadSheet()   - 
Constructor for class bioroot.strain. StrainSpreadSheet 
  
   StrainUpload   - Class in  bioroot.strain      StrainUpload()   - 
Constructor for class bioroot.strain. StrainUpload 
  
   StrainUploadBean   - Class in  bioroot.strain  Strain bulk file upload bean.   StrainUploadBean()   - 
Constructor for class bioroot.strain. StrainUploadBean 
  
   StrainViewPreferences   - Class in  bioroot.strain      StrainViewPreferences()   - 
Constructor for class bioroot.strain. StrainViewPreferences 
  
   STRAND_TAG   - 
Static variable in class edu.utah.seq.parsers. BarParser 
 Reserved tag names for writing to bar file tagValue pairs.
   stringArrayListToString(ArrayList, String)   - 
Static method in class selex. SeqFiles 
 Returns a String separated by the separator given an ArrayList of String.
   stringArrayListToString(ArrayList, String)   - 
Static method in class util.gen. Misc 
 Returns a String separated by the separator given an ArrayList of String.
   stringArrayListToStringArray(ArrayList)   - 
Static method in class util.gen. Misc 
 Returns a String[] given an ArrayList of Strings.
   stringArrayToArrayList(String[])   - 
Static method in class selex. SeqFiles 
 Returns an ArrayList of String given a String[]
   stringArrayToArrayList(String[])   - 
Static method in class util.gen. Misc 
 Returns an ArrayList of String given a String[]
   stringArrayToFloat(String, String)   - 
Static method in class util.gen. Num 
 Given a String of floats delimited by something, will parse or return null.
   stringArrayToInts(String, String)   - 
Static method in class util.gen. Num 
 Given a String of ints delimited by something, will parse or return null.
   stringArrayToString(String[], String)   - 
Static method in class util.gen. Misc 
 Returns a String separated by commas for each bin.
   stringFeatures   - 
Static variable in class bioroot.antibody. AntibodyBase 
 Use to make a default antibody Feature[].
   stringFeatures   - 
Static variable in class bioroot.oligo. OligoBase 
 Use to make a default oligo Feature[].
   stringFeatures   - 
Static variable in class bioroot.plasmid. PlasmidBase 
 Use to make a default plasmid Feature[].
   stringFeatures   - 
Static variable in class bioroot.strain. StrainBase 
 Use to make a default strain Feature[].
   stringRep(int)   - 
Method in class trans.main. Window 
 Returns chromosome, start first oligo, start last oligo
   stripIt(Pattern, Pattern, Pattern, String)   - 
Static method in class util.bio.parsers.gff. DmelRel4Extractor 
  
   StripZeros   - Class in  expr      StripZeros(String[])   - 
Constructor for class expr. StripZeros 
  
   subArray(float[][], int, int)   - 
Static method in class util.gen. Num 
 Builds a sub array from part of a float[replica][intensities].
   submitNew(DBUtil)   - 
Method in class bioroot.antibody. AntibodyBean 
 Makes a new Antibody table entry, don't forget to save associated strains and plasmids after
 updating this bean with its new id number.
   submitNew(DBUtil)   - 
Method in class bioroot.antibody. AntibodyPrep 
 Makes a new AntibodyPrep table entry.
   submitNew(DBUtil, int)   - 
Method in class bioroot.antibody. UseDilution 
  
   submitNew(DBUtil, UserBean)   - 
Method in class bioroot. CellTypeBean 
  
   submitNew(DBUtil, UserBean)   - 
Method in class bioroot. GeneBean 
  
   submitNew(DBUtil, UserBean)   - 
Method in class bioroot. MarkerBean 
  
   submitNew(DBUtil)   - 
Method in class bioroot.oligo. OligoBean 
  
   submitNew(DBUtil, UserBean)   - 
Method in class bioroot. OrganismBean 
  
   submitNew(DBUtil)   - 
Method in class bioroot.plasmid. PlasmidBean 
  
   submitNew(DBUtil)   - 
Method in class bioroot.strain. StrainBean 
 Makes a new Strain table entry, don't forget to save associated markers and plasmids after
 updating this bean with its new id number.
   submitNewUser()   - 
Method in class bioroot. UserRegistrationBean 
  
   subSample(HashMap<String, PointData>, int)   - 
Static method in class edu.utah.seq.data. PointData 
 Reduces the number of observations to the specified number by random selection.
   subSamplePercent(HashMap<String, PointData>[], int)   - 
Static method in class edu.utah.seq.data. PointData 
 Will draw the numPos wanted from the array of PointData sampling randomly within but equally throughout.
   SubSamplePointData   - Class in  edu.utah.seq.data      SubSamplePointData(String[])   - 
Constructor for class edu.utah.seq.data. SubSamplePointData 
 Randomly samples out reads to match a desired number.
   SubSeq   - Class in  selex  Holds information related to a particular parsed SELEX oligo.   SubSeq(String, int[], SelexParams, SeqRead)   - 
Constructor for class selex. SubSeq 
  
   subtract(Coordinate, Coordinate)   - 
Static method in class util.bio.annotation. Coordinate 
 Clips the forReduction based on the mask.
   subtractAndSetAntibodyBeans(AntibodyBean[], HttpSession)   - 
Static method in class bioroot.antibody. AntibodyModify 
 Subtracts a bean from the AntibodyBean[] and resets it in antibodyBeansToEdit
   subtractAndSetOligoBeans(OligoBean[], HttpSession)   - 
Static method in class bioroot.oligo. OligoModify 
 Subtracts a bean from the OligoBean[] and resets it in oligoBeansToEdit
   subtractAndSetPlasmidBeans(PlasmidBean[], HttpSession)   - 
Static method in class bioroot.plasmid. PlasmidModify 
 Subtracts a bean from the PlasmidBean[] and resets it in plasmidBeansToEdit
   subtractAndSetStrainBeans(StrainBean[], HttpSession)   - 
Static method in class bioroot.strain. StrainModify 
 Subtracts a bean from the StrainBean[] and resets it in strainBeansToEdit
   subtractIntervals(Interval[], Interval[])   - 
Static method in class trans.main. OverlapCounter 
 Returns the difference between two interval arrays, be sure to get bigger and smaller correct.
   SubtractOneFromEachColumn   - Class in  util.apps  Subtracts 1 from each column assumes integers.   SubtractOneFromEachColumn()   - 
Constructor for class util.apps. SubtractOneFromEachColumn 
  
   subtractOneFromFeatures()   - 
Method in class util.bio.parsers.gff. Gff3Parser 
 Subract one from start and end of each feature.
   SubtractRegions   - Class in  trans.anno  Intersects two lists of regions subtracting the first from the second.   SubtractRegions(String[])   - 
Constructor for class trans.anno. SubtractRegions 
  
   SubWindow   - Class in  trans.main  Class to hold information about a subWindow.   SubWindow(int, double)   - 
Constructor for class trans.main. SubWindow 
  
   sumArray(float[])   - 
Static method in class util.gen. Num 
 Sums a float array
   SUMC_ID   - 
Static variable in class edu.utah.seq.analysis. ScanSeqs 
  
   sumIntArray(int[])   - 
Static method in class util.gen. Num 
 Sums an int array
   summaryLine()   - 
Method in class trans.anno. BindingRegion 
 Returns tab delimited line: rank, chrom, start, end, dist to closes gene, gene name(s), # neighbors
   summaryLine()   - 
Method in class trans.main. PosRegion 
 Returns tab delimited line: rank, chrom, start, end, dist to closes gene, gene name(s), # neighbors
   sumPositionBP(int, int)   - 
Method in class edu.utah.seq.data. PointData 
 Given a start bp (included) and stop bp (not included), returns the sum of the associate positions, the hit count.
   sumRanks()   - 
Method in class trans.main. WilcoxonRankSumTest 
  
   sumScoreBP(int, int)   - 
Method in class edu.utah.seq.data. PointData 
 Given a start bp (included) and stop bp (not included), returns the sum of the associate scores.
   sumScoreIndex(int, int)   - 
Method in class edu.utah.seq.data. PointData 
 Given a start index (included) and stop index (NOT included), returns the sum of the associate scores.
   SUMT_ID   - 
Static variable in class edu.utah.seq.analysis. ScanSeqs 
  
   sumTargetScores()   - 
Method in class expr. ParseMiRNATable 
  
   sumWindowScores(int, int, QCSeqs.ChromWindowScores)   - 
Method in class edu.utah.seq.analysis. QCSeqs 
  
   Swing   - Class in  util.gen  Methods related to Swing apps.   Swing()   - 
Constructor for class util.gen. Swing 
  
   SynonymMatching   - Class in  util.bio.parsers      SynonymMatching(String[])   - 
Constructor for class util.bio.parsers. SynonymMatching 
  
 
 


 
   
  
 
 
 
   
 
   
          Overview     
        Package   
        Class   
        Use   
          Tree     
          Deprecated     
       Index    
          Help     
   
 
 
  
 
 
 

 
  
   PREV LETTER   
   NEXT LETTER    
  
    FRAMES     
   NO FRAMES     
  
    All Classes  ');
  }
  //-->
 
 
    All Classes  
 


  
 
 
  
 

 A   B   C   D   E   F   G   H   I   J   L   M   N   O   P   Q   R   S   T   U   V   W   X   Z
